# Characterization of two MHC II genes (DOB, DRB) in white-tailed deer (*Odocoileus virginianus*)

**DOI:** 10.1186/s12863-020-00889-5

**Published:** 2020-07-29

**Authors:** Natascha M. D. Ivy-Israel, Carolyn E. Moore, Tonia S. Schwartz, Stephen S. Ditchkoff

**Affiliations:** 1 School of Forestry and Wildlife Sciences, Auburn University, Auburn, AL 36849 USA; 2 Department of Biological Sciences, Auburn University, Auburn, AL 36849 USA

**Keywords:** Major histocompatibility complex, Linkage disequilibrium, *Odocoileus virginianus*, Ruminant, Chromosomal inversion, MiSeq

## Abstract

**Background:**

The major histocompatibility complex (MHC) is responsible for detecting and addressing foreign pathogens inside the body. While the general structure of MHC genes is relatively well conserved among mammalian species, it is notably different among ruminants due to a chromosomal inversion that splits MHC type II genes into two subregions (IIa, IIb). Recombination rates are reportedly high between these subregions, and a lack of linkage has been documented in domestic ruminants. However, no study has yet examined the degree of linkage between these subregions in a wild ruminant. The white-tailed deer (*Odocoileus virginianus*), a popular ruminant of the Cervidae family, is habitually plagued by pathogens in its natural environment (e.g. *Haemonchus contortus*, *Elaeophora*). Due to the association between MHC haplotypes and disease susceptibility, a deeper understanding of MHC polymorphism and linkage between MHC genes can further aid in this species’ successful management. We sequenced *MHC-DRB* exon 2 (IIa) and *MHC-DOB* exon 2 (IIb) on the MiSeq platform from an enclosed white-tailed deer population located in Alabama.

**Results:**

We identified 12 new *MHC-DRB* alleles, and resampled 7 alleles, which along with other published alleles brings the total number of documented alleles in white-tailed deer to 30 for *MHC-DRB* exon 2. The first examination of *MHC-DOB* in white-tailed deer found significantly less polymorphism (11 alleles), as was expected of a non-classical MHC gene. While *MHC-DRB* was found to be under positive, diversifying selection, *MHC-DOB* was found to be under purifying selection for white-tailed deer. We found no significant linkage disequilibrium between *MHC-DRB* and *MHC-DOB*, suggesting that these loci are unlikely to be closely linked.

**Conclusions:**

Overall, this study identified 12 new *MHC-DRB* exon 2 alleles and characterized a new, non-classical, MHC II gene (*MHC-DOB*) for white-tailed deer. We also found a lack of significant linkage between these two loci, which supports previous findings of a chromosomal inversion within the MHC type II gene region in ruminants, and suggests that white-tailed deer may have a recombination hotspot between these MHC regions similar to that found for *Bos taurus*.

## Background

The major histocompatibility complex (MHC) is a well-studied group of genes whose protein products are responsible for recognizing and addressing foreign pathogens present in the body [38, 43, 86]. While type I MHC genes are present on all nucleated cells, type II MHC genes occur only on immune cells, such as dendritic cells and macrophages [40]. These class II MHC molecules consist of two membrane-spanning chains (α and β), both of which are produced by MHC genes [105]. Immune cells, also known as antigen presenting cells (APC), engulf exogenous particles. Once broken down, the peptide fragments are bound to MHC gene products (*MHC-DR* and *MHC-DQ*), which present them at the surface of the APC. To achieve this, non-classical MHC genes, such as *MHC-DM* and *MHC-DO*, produce accessory proteins used to effectively load antigens onto classical, peptide-binding MHC gene products, which then travel to the APC’s surface to present the antigen to the immune system [40, 54, 72]. If an antigen is recognized as foreign, helper T cells will bind to the presented antigen and release lymphokines, which attract other cells to the area. These helper T cells will also bind to B cell lymphocytes, which will ultimately stimulate the production of antibodies for this particular antigen [40]. The MHC is therefore a crucial component of a vertebrate’s immune system.

The peptide binding region (PBR) determines which of the exogenous peptide fragments the MHC proteins can bind. These regions are often found to be under positive diversifying selection [10, 88], increasing the probability the gene products from different alleles of an MHC gene can bind different antigens. Through this differential antigen presentation, some allele combinations (haplotypes) can confer greater susceptibility or resistance to certain pathogens. While this is strongly documented among humans [2, 22, 32, 34, 66] and domesticated animals [48, 70, 92], it is an emerging field among wildlife species.

While the synteny of MHC genes is relatively well conserved among mammalian species [103], it is notably different among ruminants (*Bos taurus*, [6]; *Ovis aries*, [29]). More specifically, while the MHC II genes of most placental mammals are organized as centromere – (~ 20 Mb of non-MHC DNA) – *MHC-DM*/*DO* – *MHC-DQ*/*DR* – MHC III/I genes [31, 103], the ruminant MHC II genes are organized as centromere – *MHC-DM*/*DO* – (~ 20 Mb of non-MHC DNA) – *MHC-DQ*/*DR* – MHC III/I genes [16, 82]. The unique organization of MHC II genes found among ruminants is thought to be due to a chromosomal inversion in an ancestral mammal [8] that has split MHC II genes into two subregions: IIa (*MHC-DR*/*DQ*) and IIb (*MHC-DM*/*DO*/*DY*, *TAP*, among others). A similar MHC II gene configuration was found in the finless Yangtze porpoise (*Neophocaena asiaeorientalis asiaeorientalis*, [83]), but not in swine (*Sus scrofa*, [77]), which suggests the inversion occurred after the phylogenetic split between ruminants and *Suidae* but before ruminants split from *Cetacea*. The ruminant MHC II subregions are separated by at least 15 cM (centimorgans; [6, 101]), which is markedly greater than what is found in humans (~ 3 cM; [98]) and mice (~ 1.5 cM; *Mus musculus*, [95]). Significant recombination rates have been observed in the interval between *MHC-DR* and *MHC-DY* genes in *Bos taurus* [68, 69], further suggesting a recombination hotspot between the two subregions in ruminants.

Studies on deer species have found the *MHC-DR* and *MHC-DQ* genotypes to be important for disease resistance. Li et al. [50] found that one *MHC-DR*/*DQ* haplotype was associated with resistance to purulent disease, a multifactorial disease [51], among forest musk deer (*Moschus berezovskii*), whereas two other haplotypes increased susceptibility to the disease. Different *MHC-DR* haplotypes in Iberian red deer (*Cervus elaphus hispanicus*) influenced susceptibility to a variety of different pathogens [27]. For example, while one haplotype was associated with a decreased occurrence of tuberculosis but increased *Elaphostrongylus cervi* scores, deer with a different haplotype experienced the opposite trend. Similarly, when using phylogenetic groupings of *MHC-DRB* exon 2 alleles as haplotypes, Ditchkoff et al. [24] found that white-tailed deer (*Odocoileus virginianus*) with different haplotypes experienced different levels of abomasal nematodes (including *Haemonchus contortus*) and ectoparasitism by ticks.

White-tailed deer are a well-studied wild ruminant due to their popularity as a game species [39]. While proper management has significantly improved their numbers, they are continually threatened by pathogens such as *Haemonchus contortus* [73, 74], *Elaeophora* [19], and the epizootic hemorrhagic disease virus and bluetongue virus [28]. These diseases can have significant impacts on deer populations and a deeper understanding of factors that influence susceptibility to these diseases is crucial to managing their populations. While the *MHC-DRB* region has been previously characterized in white-tailed deer [99, 100], other MHC genes (such as *MHC-DOB*) and the possibility of the inverted MHC genetic configuration found among other ruminants have not. To date there are 18 documented *MHC-DRB* exon 2 alleles in white-tailed deer [99, 100]. Newer sequencing technologies (Next Generation Sequencing), however, may reveal greater polymorphism due to their greatly increased mutation detection rate (sensitivity; [14, 15, 41]). Additionally, while homologs of bovine MHC IIb genes have been identified in several species belonging to Cervidae [96], no study has directly examined the possibility of an inverted MHC II configuration in a cervid. While the white-tailed deer genome has been sequenced [87], the *MHC-DRB* and *MHC-DOB* genomic regions are found on different scaffolds that prevent a large-scale understanding of their arrangement.

To fully capture the association between MHC haplotypes and disease susceptibility, we must first have a better understanding of the polymorphism that exists at these genes. We therefore aim to further quantify *MHC-DRB* exon 2 polymorphism in white-tailed deer and characterize an additional MHC gene (*MHC-DOB* exon 2). Since these genes are predicted to lie on different MHC II subregions separated by the inversion seen in other ruminants, we also tested for linkage disequilibrium between *MHC-DRB* and *MHC-DOB* in white-tailed deer. Since the immunological functions of *MHC-DRB* and *MHC-DOB* are vastly different, with *MHC-DRB* being the classical antigen-presenting protein and *MHC-DOB* being an accessory loading protein, selection may have influenced these genes differently. We therefore also assessed how these two genes have evolved to better understand selection pressures on MHC polymorphism of white-tailed deer.

## Results

### Sequencing

Before filtering, the average number of reads for individuals was 30,549 for *MHC-DRB* (ranged from 5318 to 84,226 reads with a median of 24,309) and 22,341 for *MHC-DOB* (ranged from 7029 to 49,892 reads with a median of 21,913). The number of reads recovered per individual after filtering for quality ranged from 4162 to 66,582 reads for *MHC-DRB* (average = 24,795.5; median = 19,214) and from 2779 to 36,407 reads for *MHC-DOB* (average = 15,815; median = 15,522). While unfiltered read lengths ranged from 289 to 488 bp for *MHC-DRB* (average = 306, median = 307) and from 50 to 535 bp for *MHC-DOB* (average = 397, median = 398), filtered read lengths were 296 to 365 bp long for *MHC-DRB* (average = 306, median = 307) and 329 to 406 bp long for *MHC-DOB* (average = 397, median = 398). All raw sequence data is available on the NCBI Sequence Read Archive (SRA accession # PRJNA533917).

### No linkage disequilibrium between *MHC-DRB* and *MHC-DOB*

We found no significant linkage disequilibrium between *MHC-DRB* exon 2 and *MHC-DOB* exon 2 when using only the individuals in the starting population (*n* = 69; *p* = 0.95) and when including individuals without pedigree data to this initial population (*n* = 122; *p* = 0.37). Since these loci are unlikely to be closely linked, we present the results for each gene separately.

### *MHC-DRB*

#### Defined 12 new alleles

A total of 19 *MHC-DRB* exon 2 alleles were found in our white-tailed deer population (*n* = 373), 12 of which were new alleles (Odvi-DRB*19 – Odvi-DRB*30; Table S2). The seven previously identified alleles found in the population were Odvi-DRB*01, Odvi-DRB*05, Odvi-DRB*06, Odvi-DRB*10, Odvi-DRB*12, Odvi-DRB*14, and Odvi-DRB*16 [99, 100]. The 19 *MHC-DRB* exon 2 alleles found in our population translated to 18 unique amino acid alleles (82–83 codons), as Odvi-DRB*06 and Odvi-DRB*19 differed by one synonymous substitution (Table S3). Together with the other 12 previously identified *MHC-DRB* exon 2 alleles [99, 100] not recovered in our population, a total of 30 *MHC-DRB* exon 2 alleles have been characterized for white-tailed deer. The 12 new *MHC-DRB* exon 2 alleles have been deposited in Genbank under accession numbers MK952679- MK952690.

#### Genetic relationship among alleles

The length of *MHC-DRB* exon 2 alleles was 250 bp for all alleles except for Odvi-DRB*27, which had a 3-bp frameshift deletion. Pairwise nucleotide distances among alleles ranged from 0.40 to 18.40% (mean = 10.99%), and pairwise amino acid distances ranged from 0 to 39.35% (mean = 23.00%). The average number of nucleotide differences (k) was 24.97, and there were 85 polymorphic sites in the *MHC-DRB* exon 2 sequences.

**Fig. 1:**
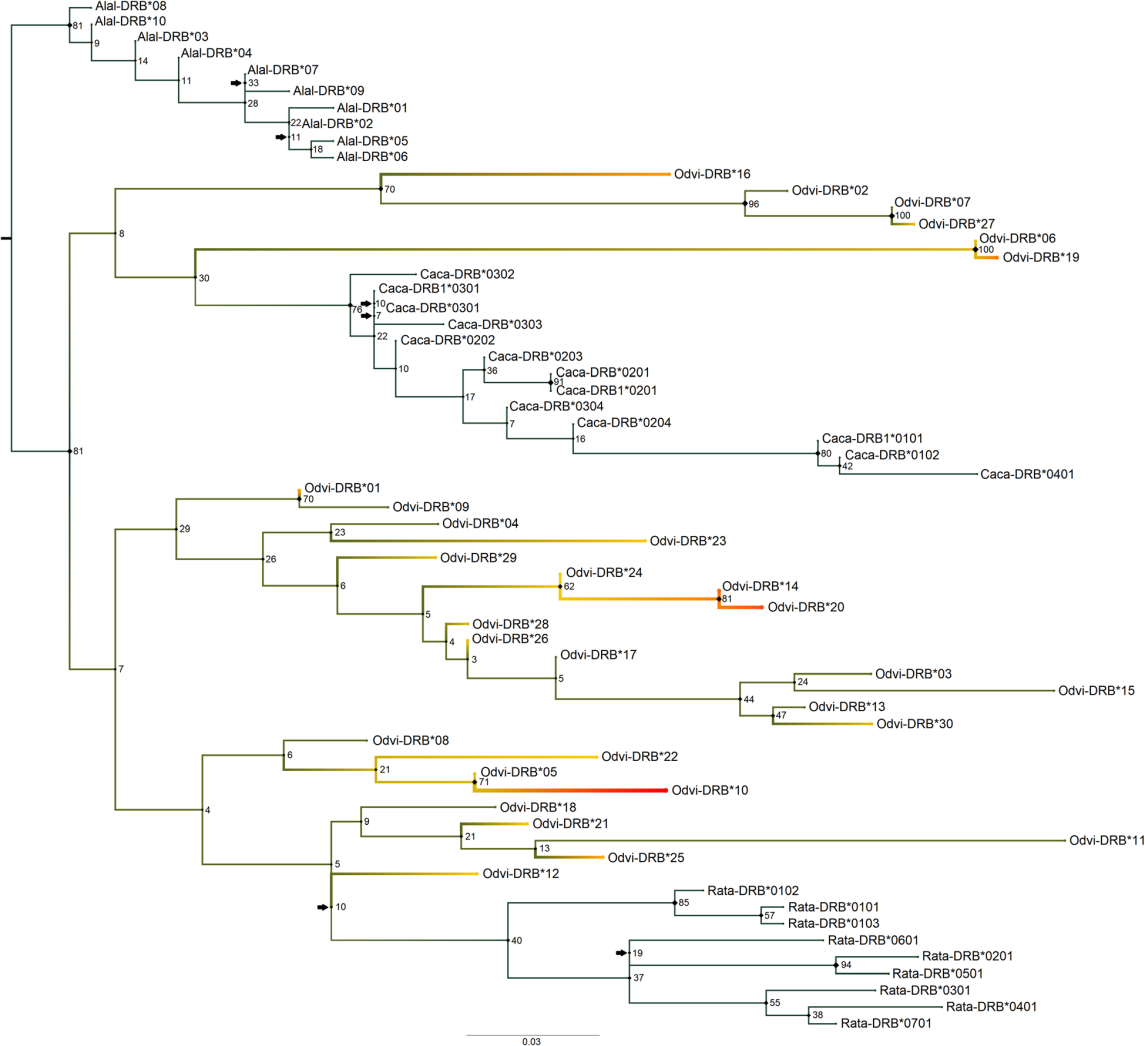
Maximum likelihood phylogenetic tree for the nucleotide sequences of *MHC-DRB* exon 2. This tree contains sequences for white-tailed deer (Odvi, *Odocoileus virginianus*; AF082161-AF082175, AF407169-AF407171, MK952679- MK952690), moose (Alal, *Alces alces*; X82398, X83278, X83279-X83286; [56]), roe deer (Caca, *Capreolus capreolus*; KM488213-KM488216, KM488218, KM488220-U90925; [57, 75]), and reindeer (Rata, *Rangifer tarandus*; AF012716-AF012724; [59]) as these are the closest related species to white-tailed deer with *MHC-DRB* data. It was rooted with the moose outgroup. Node labels are standard bootstrap support (%). Arrows indicate the presence of near-zero internal branch lengths (< 0.0040), which should be interpreted with caution. Heatmap colors indicate all white-tailed deer *MHC-DRB* exon 2 alleles and further correspond to Odvi-DRB allele frequencies found in our population, where red is the most common *MHC-DRB* allele

**Fig. 2:**
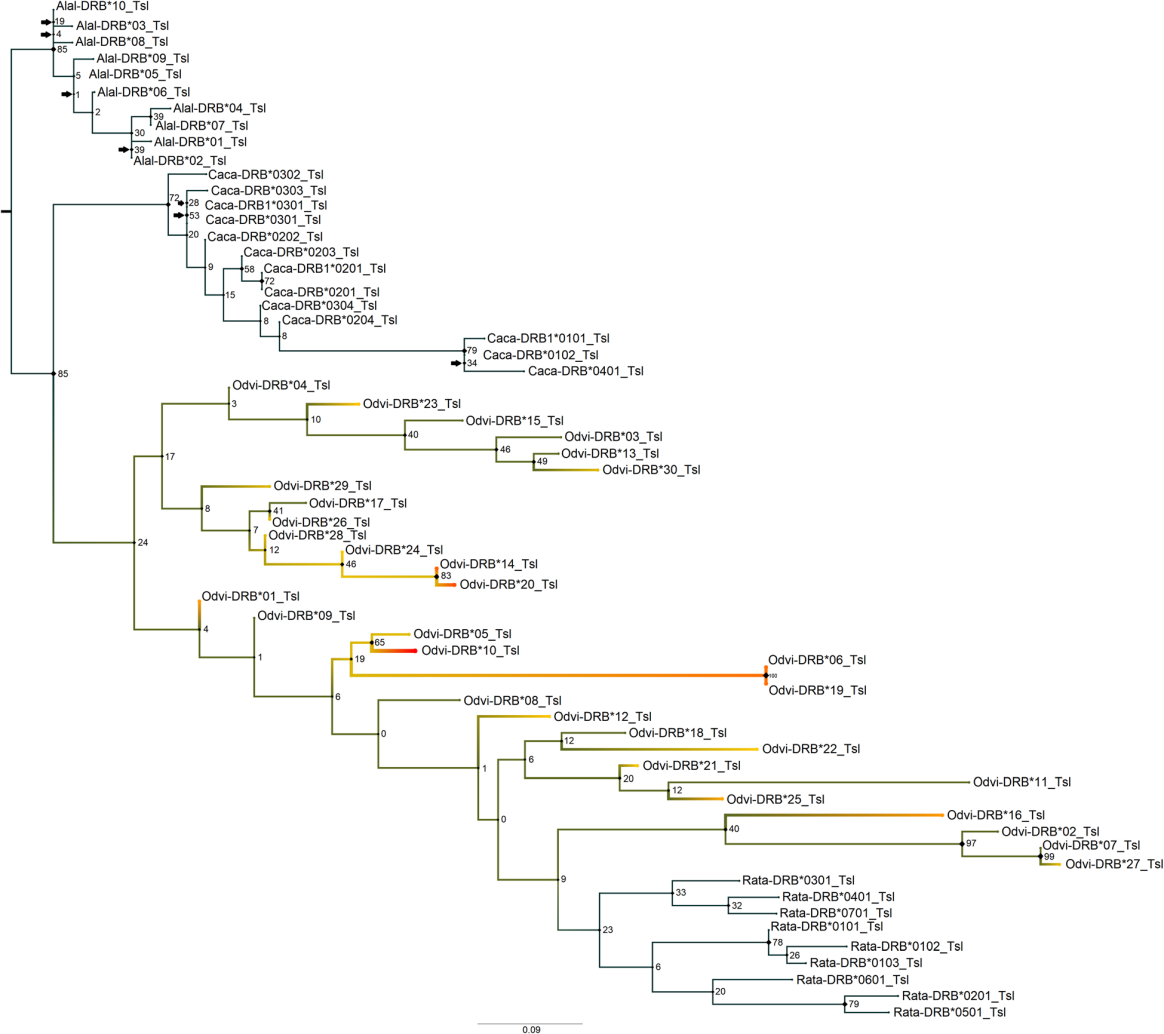
Maximum likelihood phylogenetic tree for the amino acid sequences of *MHC-DRB* exon 2. This tree contains the translated sequences for white-tailed deer (Odvi, *Odocoileus virginianus*; AF082161-AF082175, AF407169-AF407171, MK952679- MK952690), moose (Alal, *Alces alces*; X82398, X83278, X83279-X83286; [56]), roe deer (Caca, *Capreolus capreolus*; KM488213-KM488216, KM488218, KM488220-U90925; [57, 75]), and reindeer (Rata, *Rangifer tarandus*; AF012716-AF012724; [59]) as these are the closest related species to white-tailed deer with *MHC-DRB* data. It was rooted with the moose outgroup. Node labels are standard bootstrap support (%). Arrows indicate the presence of near-zero internal branch lengths (< 0.012), which should be interpreted with caution. Heatmap colors indicate all white-tailed deer *MHC-DRB* exon 2 alleles and further correspond to Odvi-DRB allele frequencies found in our population, where red is the most common translated *MHC-DRB* allele

In assessing the phylogenetic relationships using ModelFinder, the best-fit model according to BIC for the nucleotide *MHC-DRB* tree was F81 + F + I + G4 (Fig. 1). There were six near-zero internal branches (< 0.0040): two in the moose outgroup, two in the roe deer outgroup, one in the reindeer outgroup, and one in the internal branch that separates the reindeer clade from the white-tailed deer sequences. The white-tailed deer/roe deer/reindeer *MHC-DRB* exon 2 nucleotide sequences separated fairly strongly from the moose outgroup (81% bootstrap support) but not from each other, suggesting the presence of a polytomy. While the roe deer and reindeer sequenced clustered into clear groups, the white-tailed deer sequences did not. However, there were some well-supported clades among these white-tailed deer sequences (up to 100% bootstrap support). When using translated *MHC-DRB* exon 2 sequences, the best-fit model according to BIC values was PMB + I + G4 (Fig. 2). This tree had seven near-zero internal branches (< 0.012) in terms of bootstrap support: four in the moose outgroup and three in the roe deer outgroup. As with the nucleotide tree, the white-tailed deer/roe deer/reindeer sequences separated strongly from the moose outgroup (85% bootstrap support). The roe deer sequences were further separated with 72% bootstrap support, while the white-tailed deer and reindeer sequences remained together, though there were some well-supported clades within these remaining sequences (up to 100% bootstrap support).

#### Population genetic measures and test for selection

When assessing the allele frequencies of the complete dataset (2003–2017), the most common *MHC-DRB* exon 2 allele was Odvi-DRB*10 (22.4%), followed by Odvi-DRB*20 (14.6%) and Odvi-DRB*14 (12.5%; Fig. 1; Table S2). The most common genotypes were Odvi-DRB*20/Odvi-DRB*10 (7.0%), Odvi-DRB*10/Odvi-DRB*10 (4.8%), and Odvi-DRB*14/Odvi-DRB*10 (4.6%; Table S4). Allele frequencies changed slightly from the founding population (2003–2007) to the more recent population (2016). Frequencies for Odvi-DRB*01 (10.9–6.8%) and Odvi-DRB*14 (16.7–10.2%) decreased over time, whereas Odvi-DRB*10 (18.1–22.5%) and Odvi-DRB*19 (5.8–11.9%) became more frequent in the population. Odvi-DRB*05, a rare allele in our original founding population, was lost from the population over time. While *MHC-DRB* exon 2 was under Hardy Weinberg equilibrium using the probability-test for both the founding and 2016 populations (*p* = 0.88 and *p* = 0.91, respectively), score test results for the 2016 population indicated heterozygote excess in the population (*p* = 0.05; Table 1).

**Table 1:** Genetic diversity within our white-tailed deer population (nucleotide diversity and haplotype diversity) and neutrality tests for *MHC-DRB* exon 2 for the adult founding population [2003–2007; *n* = 69 (*MHC-DRB*), 71 (*MHC-DOB* extended sequence), 71 (*MHC-DOB* exon 2)] and the final adult population [2016; *n* = 118 (*MHC-DRB*), 119 (*MHC-DOB* extended sequence), 119 (*MHC-DOB* exon 2)]. *P*-values for the Hardy Weinberg Equilibrium tests are also included, which shows a statistical significance for heterozygote excess in *MHC-DRB* exon 2 in the 2016 population

	*MHC-DRB* founding population	*MHC-DRB* 2016 population
Diversity		
Nucleotide diversity (π)	0.09	0.10
Haplotype diversity	0.90	0.88
Neutrality		
Tajima’s D	0.92	1.47
Fu and Li’s F*	2.09*	2.45*
Fu and Li’s D*	2.39*	2.56*
Fu’s Fs	21.98	35.02
HWE		
Probability test	0.88	0.91
Heterozygote excess	0.70	0.05*
Heterozygote deficit	0.30	0.95

* *p* < 0.05

Both nucleotide (π) and haplotype diversity generally stayed the same over time (0.09 to 0.10 and 0.90 to 0.88, respectively; Table 1). All neutrality test values were positive, and there was an increasing trend from the founding population to the more recent 2016 population (Table 1), suggesting that selection on *MHC-DRB* is becoming less neutral in our population. Both Fu and Li’s D* and F* test statistics were significant (*p* < 0.02) for the founding (2.39 and 2.09, respectively) and 2016 (2.56 and 2.45, respectively) populations. These positive results suggest *MHC-DRB* had an excess of intermediate frequency alleles in our population, which is an indication of balancing selection and/or reduced population size. *MHC-DRB* showed evidence of positive selection based on the dN/dS ratio of 2.06 (Fig. S1).

### *MHC-DOB*

#### Defined 11 alleles

Eleven unique alleles were identified for the extended *MHC-DOB* nucleotide sequences (Odvi-DOB*01 – Odvi-DOB*11) and seven alleles for *MHC-DOB* exon 2 in our white-tailed deer population (Table S5). Odvi-DOB*01, Odvi-DOB*02, and Odvi-DOB*11 contained the same exon 2 sequence (Odvi-DOB*010211_exon2). Odvi-DOB*03 and Odvi-DOB*10 also contained the same exon 2 (Odvi-DOB*0310_exon2), as well as Odvi-DOB*06 and Odvi-DOB*07 (Odvi-DOB*0607_exon2). Odvi-DOB*04, Odvi-DOB*05, Odvi-DOB*08, and Odvi-DOB*09 had unique exon 2 sequences (Odvi-DOB*04_exon2, Odvi-DOB*05_exon2, Odvi-DOB*08_exon2, Odvi-DOB*09_exon2, respectively). The seven *MHC-DOB* exon 2 alleles translated into three unique amino acid alleles (89 codons). The *MHC-DOB* alleles have been deposited in Genbank for the extended DOB sequences (accession numbers MK952691- MK952701).

#### Genetic relationships among alleles

**Fig. 3:**
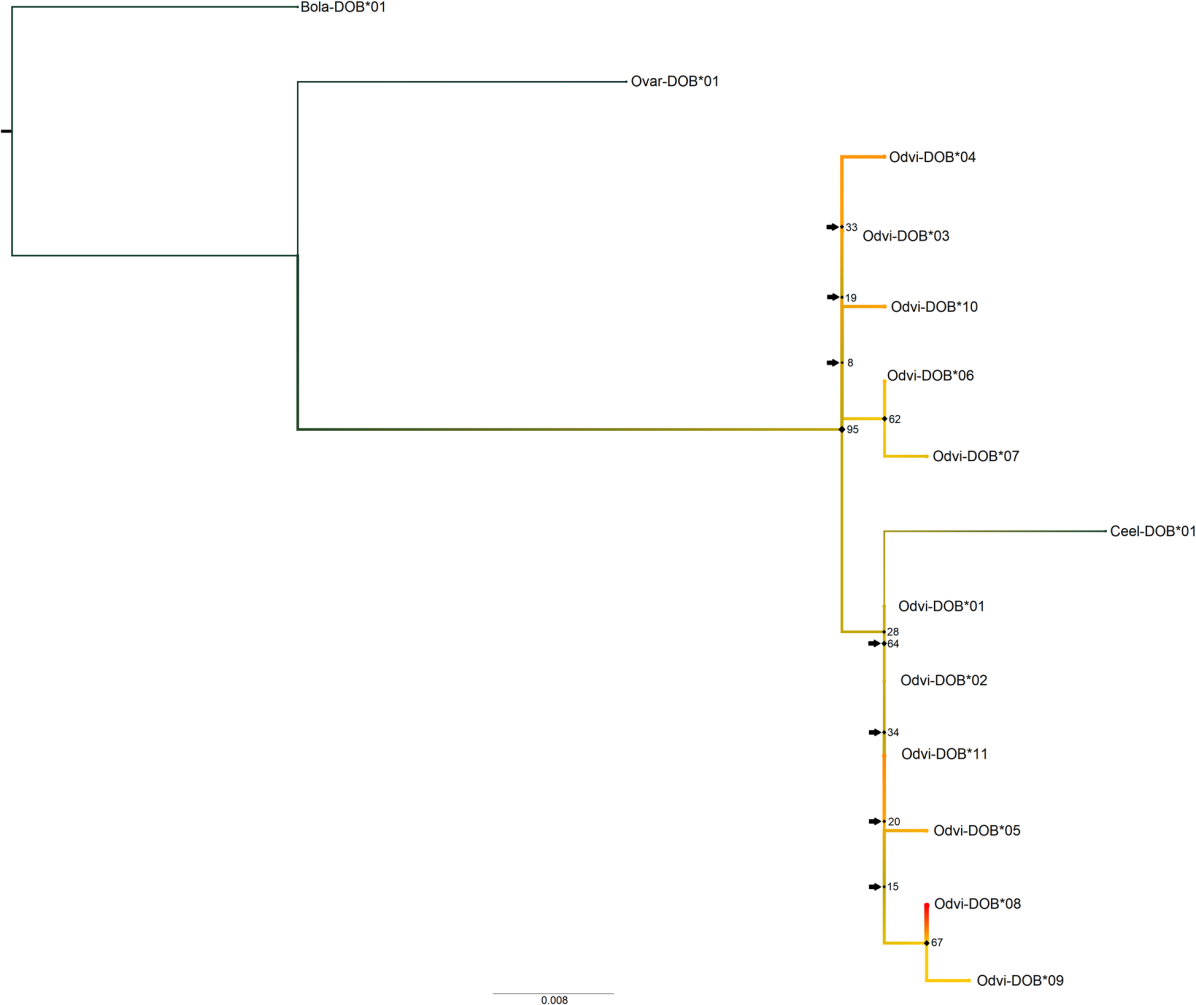
Maximum likelihood phylogenetic tree for the extended nucleotide sequences of *MHC-DOB* (360 bp). This tree contains sequences for white-tailed deer (Odvi, *Odocoileus virginianus*; MK952691- MK952701), cow (Bola, *Bos taurus*; 282493; [112]), sheep (Ovar, *Ovis aries*; Z49879.1; [107]), and red deer (Ceel, *Cervus elaphus*; CM008014.1; [7]). It was rooted with the cow outgroup. Node labels are standard bootstrap support (%). Arrows indicate the presence of near-zero internal branch lengths (< 0.0028), which should be interpreted with caution. Heatmap colors indicate all white-tailed deer *MHC-DOB* alleles and further correspond to Odvi-DOB allele frequencies found in our population, where red is the most common *MHC-DOB* allele

**Fig. 4:**
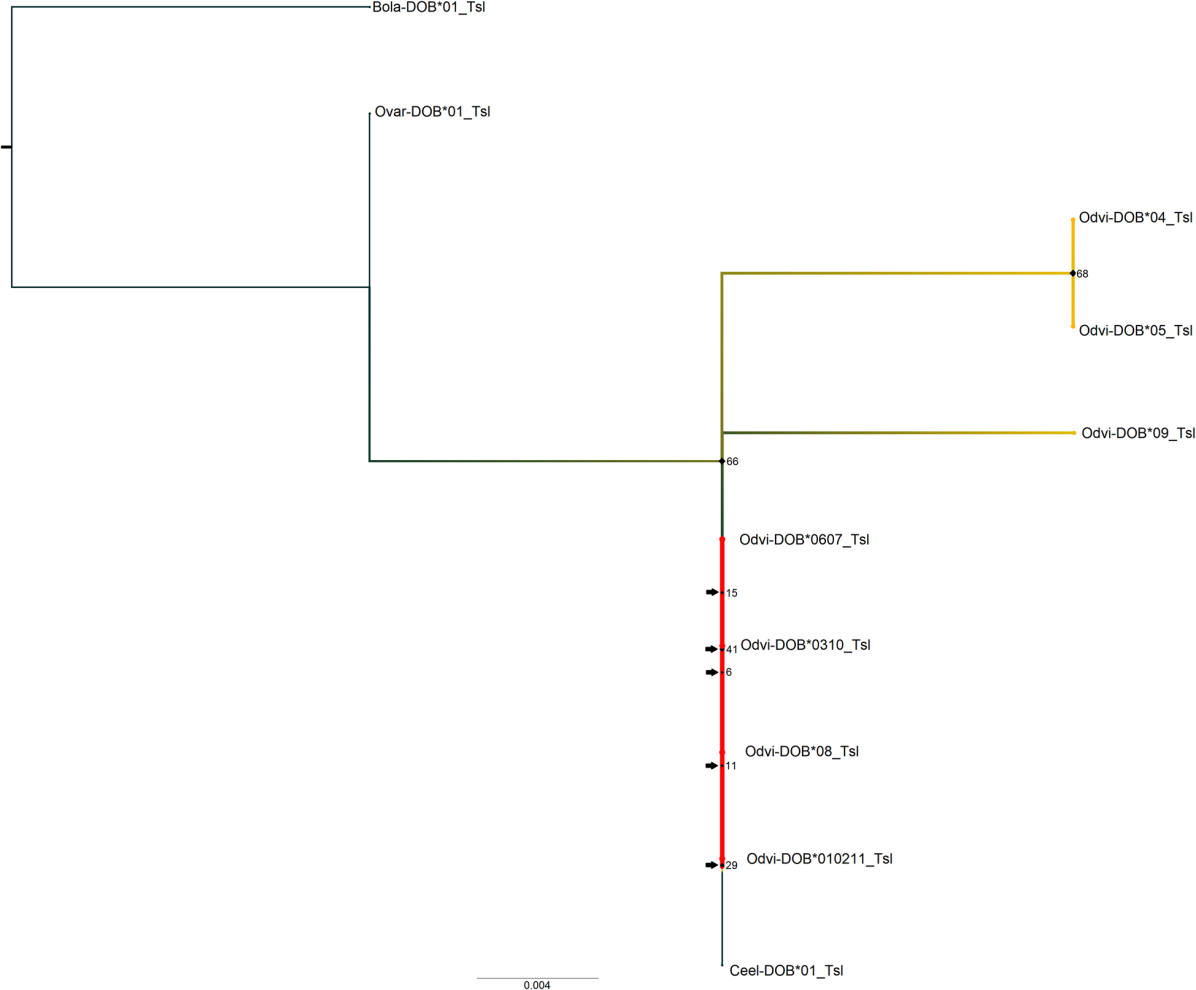
Maximum likelihood phylogenetic tree for the amino acid sequences of *MHC-DOB* exon 2. This tree contains translated sequences for white-tailed deer (Odvi, *Odocoileus virginianus*; MK952691- MK952701), cow (Bola, *Bos taurus*; 282493; [112]), sheep (Ovar, *Ovis aries*; Z49879.1; [107]), and red deer (Ceel, *Cervus elaphus*; CM008014.1; [7]). It was rooted with the cow outgroup. Node labels are standard bootstrap support (%). Arrows indicate the presence of near-zero internal branch lengths (< 0.011), which should be interpreted with caution. Heatmap colors indicate all white-tailed deer *MHC-DOB* alleles and further correspond to Odvi-DOB allele frequencies found in our population, where red is the most common translated *MHC-DOB* allele for *MHC-DOB* exon 2 in white-tailed deer, though both are quite rare

In assessing the phylogenetic relationships using ModelFinder, the best-fit model according to BIC for the nucleotide *MHC-DOB* tree was K2P + I (Fig. 3). There were seven near-zero internal branches (< 0.0028%) in terms of bootstrap support, all of which occurred in the white-tailed deer clades. The red deer and white-tailed deer sequences separated strongly from the cow and sheep sequences (97% bootstrap support). Odvi-DOB*08/Odvi-DOB*09 and Odvi-DOB*06/Odvi-DOB*07 separated from the other white-tailed deer sequences with 67 and 62% bootstrap support, respectively. The translated *MHC-DOB* tree had Blosum62 as the best-fit model (Fig. 4). There were five near-zero internal branches (< 0.0112%), which all occurred in the white-tailed deer clades. Apart from the fairly strong separation between cow/sheep sequences and the white-tailed deer/red deer sequences (66% bootstrap support), the amino acid sequences for *MHC-DOB* exon 2 did not retain the same organization as in the *MHC-DOB* nucleotide tree. The tree clearly demonstrates the three unique *MHC-DOB* exon 2 translations.

All extended *MHC-DOB* sequence alleles were 360 bp in length except for Odvi-DOB*01 (359 bp) and Odvi-DOB*02 (361 bp) due to an indel in the intronic region. The pairwise nucleotide distances ranged from 0 to 1.41% (mean = 0.58%). Odvi-DOB*01, Odvi-DOB*02, and Odvi-DOB*11 differed by one indel located in a highly repetitive region of the intron. The greatest amount of dissimilarity was found between Odvi-DOB*07 and Odvi-DOB*09 (4 synonymous substitutions, one nonsynonymous substitution; Table S6). The average number of nucleotide differences (k) was 2.07, and the extended *MHC-DOB* sequences had seven polymorphic sites. The extended nucleotide *MHC-DOB* sequences were in Hardy Weinberg Equilibrium for both the founding and 2016 populations (probability test; *p* = 0.27 and *p* = 0.55, respectively), and there was no indication of either a heterozygote deficit or excess in the founding population (Table 2). However, there was a heterozygote deficit in the 2016 population (score test; 0.03).

**Table 2:** Genetic diversity within our white-tailed deer population (nucleotide diversity and haplotype diversity) and neutrality tests for *MHC-DOB* for the adult founding population [2003–2007; *n* = 71 (*MHC-DOB* extended sequence, 360 bp), 71 (*MHC-DOB* exon 2, 270 bp)] and the final adult population [2016; *n* = 119 (*MHC-DOB* extended sequence, 360 bp), 119 (*MHC-DOB* exon 2, 270 bp)]. *P*-values for the Hardy Weinberg Equilibrium tests are also included, which shows a statistical significance for heterozygote deficit in *MHC-DOB* (extended sequence and exon 2) in the 2016 population

	Extended ***MHC-DOB*** Sequence		***MHC-DOB*** exon 2	
	Founding population	2016 population	Founding population	2016 population
Diversity				
Nucleotide diversity (π)	0.005	0.005	0.006	0.006
Haplotype diversity	0.83	0.86	0.80	0.83
Neutrality				
Tajima’s D	0.87	1.31	1.38	1.77^
Fu and Li’s F*	1.26	1.42	1.34	1.48^
Fu and Li’s D*	1.17	1.13	1.01	0.97
Fu’s Fs	−0.22	0.64	0.60	1.33
HWE				
Probability Test	0.27	0.55	0.57	0.33
Heterozygote Excess	0.77	0.97	0.44	0.99
Heterozygote Deficit	0.24	0.03*	0.57	0.01*

* *p* < 0.05; ^ 0.10 > *p* > 0.05

All alleles for the *MHC-DOB* exon 2 region contained within our extended *MHC-DOB* sequences had a length of 270 bp. The mean pairwise nucleotide distance for *MHC-DOB* exon 2 was 0.78% (0.37–1.50%), while the mean pairwise amino acid distance was 0.86% (0–2.27%). As was seen in the extended *MHC-DOB* sequences, the greatest amount of dissimilarity was found between Odvi-DOB*0607_exon2 and Odvi-DOB*09_exon2 (3 synonymous substitutions, one nonsynonymous substitution; Table S7). Odvi-DOB*04_exon2 and Odvi-DOB*09_exon2 also differed more than other *MHC-DOB* exon 2 alleles (2 synonymous substitutions, 2 nonsynonymous substitutions). The *MHC-DOB* exon2 sequences contained five polymorphic sites and 2.10 nucleotide differences on average. As with the extended *MHC-DOB* sequences, the *MHC-DOB* exon 2 alleles were also in Hardy Weinberg Equilibrium for the founding and 2016 populations (probability test; *p* = 0.57 and *p* = 0.33, respectively), though there was statistical significance for a heterozygote deficiency in the 2016 population (score test; *p* = 0.01; Table 2).

#### Population genetic measures and test for selection

Odvi-DOB*08 and Odvi-DOB*08_exon2 were the most common alleles for the extended *MHC-DOB* sequences (28.1%) and *MHC-DOB* exon 2 (27.8%; Fig. 3; Table S5). The most common genotypes all contained Odvi-DOB*08 and Odvi-DOB*08_exon2 (Table S8, S9). Allele frequencies increased over time for Odvi-DOB*06 (3.5 to 7.1%), Odvi-DOB*09_exon2 (4.2 to 9.7%), and Odvi-DOB*11/Odvi-DOB*010211_exon2 (9.2 to 12.6% and 11.3 to 14.3%, respectively), whereas Odvi-DOB*03 (12.7 to 10.5%), Odvi-DOB*05_exon2 (10.6 to 7.1%), and Odvi-DOB*08/Odvi-DOB*08_exon2 (both from 33.1 to 25.2%) became less frequent in the population.

While nucleotide diversity did not change over time, haplotype diversity increased slightly (Table 2). Neutrality test results indicated an increasing trend from the founding population to the more recent population for both extended *MHC-DOB* sequences and *MHC-DOB* exon 2 (Table 2), with Tajima’s D and Fu and Li’s F* approaching statistical significance (0.10 > *p* > 0.05) for the 2016 population. Tajima’s D increased from 0.87 to 1.31 for the extended *MHC-DOB* sequences and from 1.38 to 1.77 for *MHC-DOB* exon 2. Fu’s Fs for *MHC-DOB* was notably smaller compared to *MHC-DRB*. The dN/dS ratio for *MHC-DOB* exon 2 was 0.22, indicating no evidence of positive selection (Fig. S2).

## Discussion

In this study we aimed to characterize *MHC-DRB* exon 2 and *MHC-DOB* exon 2 in our white-tailed deer population and to examine the extent of linkage between these two loci. No significant linkage was found between the second exons of *MHC-DRB* and *MHC-DOB* in our white-tailed deer population. While further examination is needed, this finding suggests that white-tailed deer may have the same chromosomal inversion and recombination hotspot within their MHC type II gene region as found in other ruminants [4, 6, 16, 29, 68, 69]. This MHC II inversion has been documented among cetaceans [83, 84, 110] but not in swine [77], suggesting that this breakpoint may have occurred after the divergence of Cetartiodactyla and Suidae but before the divergence between Cetacea and ruminants (~ 58 million years ago; [111]). A lack of linkage disequilibrium between these two loci allows them to evolve independently from one another. Because they are therefore unlikely to be closely linked, we can contrast their evolutionary patterns as independent loci.

Our white-tailed deer population had 19 *MHC-DRB* exon 2 alleles, which is comparable to what Van Den Bussche et al. [99] found in a free-ranging white-tailed deer population in Oklahoma (15 *MHC-DRB* exon 2 alleles, *n* = 150). Even though white-tailed deer experienced severe, historical population bottlenecks [25, 49], they have managed to retain more *MHC-DRB* polymorphism than other species that survived similar population reductions, such as moose [56], bison (*Bison bison*, [58]), fallow deer (*Dama dama*) and roe deer [59]. The polymorphism found among our founding adult population was less (17 *MHC-DRB* exon 2 alleles; *n* = 69) than what was reported in unrelated individuals of red deer (34 *MHC-DRB* exon 2 alleles, *n* = 50; [97]), Kankrej cattle (*Bos indicus*; 24 *MHC-DRB* exon 2 alleles, n = 50; [9]), and the Indian water buffalo (*Bubalus bubalis*; 22 *MHC-DRB* exon 2 alleles, *n* = 25; [20]). However, white-tailed deer have greater polymorphism than both Kankrej cattle and Indian water buffalo if all 30 *MHC-DRB* exon 2 alleles found across studies in this species are used for comparison. Red deer also have two *MHC-DRB* loci whereas white-tailed deer are believed to have only one *MHC-DRB* locus [99].

While white-tailed deer already were known to have a reasonably high *MHC-DRB* polymorphism (18 alleles; [99, 100]), we identified 12 new *MHC-DRB* exon 2 alleles in our Alabama population. The original 18 *MHC-DRB* exon 2 alleles were identified using 7 populations from Oklahoma, Iowa, Tennessee, and New York. Alabama deer have an interesting ancestry due to restocking efforts between the 1940’s and 1960’s. Six white-tailed deer subspecies were used for reintroduction [3, 52], thereby creating an admixed white-tailed deer population in Alabama. Our study population was located in Tallapoosa county, which received deer from Georgia, Arkansas, and other counties in Alabama (Clarke, Marengo, Sumter; [53]). Additionally, reintroductions using Clarke county deer occurred after Michigan deer were introduced to Clarke county. Admixture may therefore be contributing to the large number of new *MHC-DRB* exon 2 alleles identified in this study. Another potential explanation for identifying additional alleles may be our use of newer sequencing technology since the white-tailed deer *MHC-DRB* was last characterized via SSCP by Van Den Bussche et al. [99, 100]. MiSeq has greater sensitivity relative to SSCP [14, 15, 41], which may have enabled us to detect more nucleotide polymorphisms in the *MHC-DRB* exon 2 sequences.

While the outgroup sequences in our *MHC-DRB* trees clustered together as monophyletic groupings, as is expected for species with reduced MHC allelic diversity due to severe population bottlenecks [56, 59], white-tailed deer *MHC-DRB* alleles represented paraphyletic groups. This suggests that *MHC-DRB* polymorphism is most likely greater in white-tailed deer than moose, roe deer and reindeer. Additionally, the lack of separation into geographically structured phylogenetic clades suggests that there is no, or very little, relationship between genetic and geographic distance for white-tailed deer, similar to findings reported by Van Den Bussche et al. [100] and Leberg et al. [49]. Reintroductions may have weakened these geographic associations between *MHC-DRB* exon 2 alleles. When comparing our *MHC-DRB* nucleotide tree to the neighbor-joining tree constructed by Van Den Bussche et al. [99, 100], we find little similarity. This is most likely due to differences in tree construction, outgroups, and increased number of Odvi-DRB alleles included in the analyses. However, no quantification of support was provided for their trees, making a direct comparison more challenging as the level of confidence in their tree splits is unknown.

The *MHC-DOB* tree used sequences from less closely related species (cow, sheep, red deer) due to a current lack of *MHC-DOB* sequence data for Cervidae, which may have influenced tree inference. The extremely low *MHC-DOB* polymorphism found within our white-tailed deer population relative to *MHC-DRB* polymorphism may contribute to the lack of separation seen between the white-tailed deer *MHC-DOB* exon 2 alleles. Lastly, Ceel-DOB did not strongly separate from Odvi-DOB, which is similar to what Van Den Bussche et al. [99, 100] found for *MHC-DRB*.

When classifying individuals as homozygotes or heterozygotes for *MHC-DRB*, we found that the frequency of the second most common sequence was less than 35% but greater than 10% relative to the first allele (63:35–90:10) for 13.95% of our population. A large proportion of these individuals had either Odvi-DRB*14 or Odvi-DRB*20 as their second allele (25.0 and 38.3%, respectively), which was further validated using pedigree and Sanger data. This could be due to allelic dropout because of a mutation in the priming region of these alleles, thereby reducing their amplification efficiency relative to the first allele. Allelic dropout is fairly common in studies that use PCR and has previously been documented in MHC genes [94]. Positive diversifying selection could also be contributing to *MHC-DRB* alleles having substitutions in the primer regions.

Varying levels of *MHC-DRB* diversity have been found among ungulates. For example, the genetic distance between *MHC-DRB* alleles can range from large (cattle, bison, red deer) to negligible (moose; [59]). Within our white-tailed deer dataset, haplotype diversity (Hd) was similar between the second exons of *MHC-DRB* and *MHC-DOB*, while nucleotide diversity (π) was greater for *MHC-DRB* exon 2 than for *MHC-DOB* exon 2. Nucleotide diversity equal to 0.1 or greater is considered high [81], suggesting that the white-tailed deer MHC*-DRB* exon 2 alleles differed considerably from one another.

In evaluating potential selection on *MHC-DRB* exon 2, which encodes the antigen-binding site of DR molecules [30], at the species level, we found strong evidence of positive, diversifying selection (e.g. dN/dS ratio). Similar results were reported by De et al. [21] for white-tailed deer. Diversifying selection occurs when the amino acid diversity in a gene increases within a species over time [17, 108]. As *MHC-DRB* produces peptide-binding proteins, increasing this gene’s diversity may enable it to present a greater array of pathogenic antigens to the immune system [1, 13, 40, 93]. In evaluating potential selection within our population, the neutrality tests indicate that there is an excess of intermediate frequency *MHC-DRB* exon 2 alleles in our population, suggesting that the *MHC-DRB* alleles are being maintained in the population via balancing selection that is becoming more pronounced in our population over time as there was an increasing trend in all neutrality test values from the 2007 starting population until the 2016 adult population. Additionally, we see an excess of heterozygotes in the 2016 population, and this retention of *MHC-DRB* alleles may be driven by a heterozygote advantage that exists in our population, especially since MHC genes are codominant. But, since our population sizes for the HWE exact tests were relatively small, the heterozygote excess found in our 2016 population may also be a product of drift. Another explanation for the increasing Tajima’s D and Fu and Li’s F* and D* values found for *MHC-DRB* may be that while balancing selection acted on the population initially (before population enclosure), a reduced population size is now adding to the generation of intermediate frequency *MHC-DRB* exon 2 alleles in our population. While our selection analyses are consistent with *MHC-DRB* exon 2 being under balancing selection in our population, it is important to acknowledge the limitations of our study in both population size and the limited number of loci studied, thereby making it impossible to discount the power of drift in producing these patterns. Demographic processes such as population size decline affect the whole genome, whereas selection typically only affects the target loci and closely linked regions. Therefore, to fully assess the underlying reasons for the excess intermediate frequency *MHC-DRB* exon 2 alleles, future work would have to take a broader genome approach for our population.

In contrast, *MHC-DOB* exon 2 appears to have been under purifying selection at the species level (0 > dN/dS > 1; [11]) thereby eliminating harmful nonsynonymous substitutions from *MHC-DOB* in white-tailed deer. The intensity of purifying selection depends on how tolerant a genomic region is towards mutations, or how functionally constrained it is. DNA regions in which a mutation is likely to affect their gene product’s function tend to be more functionally constrained and have lower substitution rates. *MHC-DOB* exon 2, which encodes the extracellular domain of the *MHC-DOB* protein [5, 62], may therefore be highly constrained for the white-tailed deer species. While haplotype diversity at the nucleotide level was high in our population, most amino acid sequences for *MHC-DOB* exon 2 had little to no differences among them (mean pairwise difference was < 1%), and we only found 3 unique amino acid sequences. Neutrality tests for *MHC-DOB* were not significant, suggesting that *MHC-DOB* seems to be evolving neutrally within our white-tailed deer population. Although the pattern of the selection analyses is consistent with selection against heterozygous individuals (e.g. heterozygote deficiency in the 2016 population), it is also consistent with drift due to the small effective population size and potential inbreeding.

## Conclusions

Overall, this study identified 12 new *MHC-DRB* exon 2 alleles and characterized a new, non-classical, MHC II gene (*MHC-DOB*) for white-tailed deer. We also found a lack of significant linkage between these two loci, which suggests there may be a chromosomal inversion in the MHC II region of white-tailed deer. However, more research is required to confirm this. If a chromosomal inversion is indeed found, recombination rates between the two MHC II subregions should be examined, as rates have been found to differ between individuals [68]. Lastly, more MHC polymorphism may be found when using next generation sequencing for white-tailed deer populations from other parts of the Americas. Improving our understanding of MHC II gene structure and polymorphism in white-tailed deer will enable to us to further examine how these unique, highly polymorphic genes influence morphology, reproductive success, and overall population dynamics in white-tailed deer.

## Methods

### Study area

This study took place at the Auburn Captive Facility (ACF) located north of Camp Hill, Alabama. The facility was part of the Piedmont Agricultural Experiment Station, which is owned by Auburn University. Deer sampled during the study were enclosed in a 174-ha facility surrounded by a 2.6-m fence, which was constructed in October 2007. The deer present within the ACF during the study included the original deer that inhabited this area during the fence formation in 2007 and their subsequent offspring. The population size of the adult, founding population was 71, while the effective population size was 64.8, calculated as N_e_ = 4N_m_N_f_ / (N_m_ + N_f_), where N_m_ is the number of breeding males and N_f_ is the number of breeding females in the population [106]. Subsequent to fencing the area, deer were neither introduced nor hunted within the ACF. Population size varied annually between 100 and 120 deer. Deer had access to supplemental feed in the form of food plots, corn feeders, and ad libitum protein feeders. A creek and its tributaries were present on the property, which provided a reliable water source year-round.

### Animal handling

White-tailed deer aged 6 months and older were captured over 10 trapping seasons (October – July each year) from 2007 to 2017 via chemical immobilization. We administered a tranquilizer mixture into the deer’s hindquarter muscle with the use of cartridge fired dart guns (Pneu-Dart model 193) and 0.22 caliber blanks. After adding 4 cc of xylazine (100 mg/ml; Lloyd Laboratories, Shenandoah, IA) to a 5 mL vial of Telazol® (100 mg/ml; Fort Dodge Animal Health, Fort Dodge, IA; [60]), we loaded 2 cc of this tranquilizer mixture into a telemetry dart (2.0 cc, type C, Pneu-Dart Inc., Williamsport, PA), which contained a radio transmitter (Advanced Telemetry Systems, Inc., Isanti, MN) that allowed us to locate the sedated deer via radio telemetry [44]. Tolazine (1.5 ml/45.36 kg) was injected in the shoulder and hindquarter muscle to reverse the sedation once data collection was complete [61]. The animals used in this study were part of a long-term study on reproductive ecology of white-tailed deer [64] and were maintained as part of the study population until they died of natural causes. These methods were approved by the Auburn University Institutional Animal Care and Use Committee (2008–1417, 2008–1421, 2010–1785, 2011–1971, 2013–2372, 2014–2521, 2016–2964, and 2016–2985) and in compliance with the American Society of Mammalogists’ guidelines [91].

Deer received a unique 3-digit identification number at initial capture, which was displayed on ear tags and also freeze branded on the front shoulder and hind quarter of some individuals. Sex and age (tooth wear and replacement aging technique; [89]) were recorded and a 1-cm^2^ notch of tissue was removed from their ear for genetic analysis. This tissue sample was then stored in a − 80 °C freezer until DNA analysis could be performed in the laboratory.

### Sequencing

We used amplicon-seq to characterize *MHC-DRB* and *MHC-DOB* for 381 individuals. A modified version of *Symbiodinium* DNA isolation methodology proposed by Coffroth et al. [18] was used to extract DNA from tissue samples collected 2007–2013. DNeasy blood and tissue kits (Qiagen, Inc.) were used to extract DNA from tissue samples collected 2014–2017 and from previous samples with low DNA yield. DNA for 381 individuals were shipped to RTL Genomics (Research and Testing Laboratory, Lubbock TX, United States) for amplicon sequencing of 307 bp of the second exon of *MHC-DRB* (primers LA31 and LA32; [56, 90]) that targeted the peptide binding domain, and 398 bp of the second exon of *MHC-DOB* (Forward: 5′ - AAAGCCCTCCTCTCCAATCC - 3′; Reverse: 5′ - CCACCAAGGAGACCCCAAC - 3′). Primers for *MHC-DOB* were constructed using available genomic data for white-tailed deer (Ovir.te.1; NW_018336049.1; [87]).

At RTL Genomics, samples were amplified via a two-step process. First, the forward primer was constructed by combining the Illumina i5 sequencing primer (5′ - TCGTCGGCAGCGTCAGATGTGTATAAGAGACAG - 3′) with the specific forward primer for each *MHC-DRB* and *MHC-DOB*, and the reverse primer was created using the Illumina i7 sequencing primer (5′ - GTCTCGTGGGCTCGGAGATGTGTATAAGAGACAG - 3′) and the specific reverse primer for each *MHC-DRB* and *MHC-DOB*. Amplifications were performed in 25 μL reactions using Qiagen HotStar Taq master mix (Qiagen Inc., Valencia, CA), 1 μL of each 5 μM primer, and 1 μL of template on ABI Veriti thermocyclers (Applied Biosystems, Carlsbad, CA). Thermal profiles used for *MHC-DRB* and *MHC-DOB* were identical: 1) 95 °C for 5 min; 2) 33 cycles of 95 °C for 1 min; 3) 50 °C for 30 s; 4) 72 °C for 1 min; 5) final extension of 72 °C for 10 min; 6) 4 °C hold. Products from this first round of amplification were then added to a second PCR based on qualitatively determined concentrations. A second PCR was performed to index the sequence by individual. Primers for the second PCR used primers that were based on the Illumina Nextera PCR primers (Forward: 5′ - AATGATACGGCGACCACCGAGATCTACAC[i5index]TCGTCGGCAGCGTC - 3′; Reverse: 5′ - CAAGCAGAAGACGGCATACGAGAT[i7index]GTCTCGTGGGCTCGG - 3′) using the following thermal profile: 1) 95 °C for 5 min; 2) 10 cycles of 94 °C for 30 s; 3) 54 °C for 40 s; 4) 72 °C for 1 min; 5) final extension of 72 °C for 10 min; 6) 4 °C hold. All amplification products were visualized with eGels (Life Technologies, Grand Island, NY). The DNA extraction from one individual failed to amplify and was therefore removed from the dataset prior to sequencing. PCR products indexed by individual were pooled in equimolar. Each pool was size-selected in two rounds using SPRIselect Reagent (BeckmanCoulter, Indianapolis, IN) in a 0.75 ratio for both rounds. These size-selected pools were then quantified using the Qubit 4 Fluorometer (Life Technologies). Successfully amplified size-selected pools were loaded on an Illumina MiSeq (Illumina, Inc., San Diego, CA) 2 × 300 flow cell at 10 pM. The amplicons were then sequenced for 300 bp length paired-end reads on the Illumina MiSeq platform for a targeted minimum of 10 k reads per individual. Reads were demultiplexed based on the index and sorted to individual files.

To ensure the quality of the sequencing data, raw reads were trimmed using Trimmomatic (v0.35; LEADING 20, TRAILING 20, SLIDINGWINDOW: 6:20, MINLEN: 20; [12]). The quality of these trimmed reads was verified using FastQC (v0.11.8). Trimmed, paired-end reads were then merged using PEAR [109]. These merged reads were further filtered by removing reads with corrupt primers, merged reads shorter than 290 base pairs, and reads whose sequence only occurred once within an individual. Once primers were removed from these filtered merged reads, the amplicon size for *MHC-DRB* and *MHC-DOB* were 250 bp and 360 bp, respectively. While the *MHC-DRB* amplicon only captured exon 2, the *MHC-DOB* amplicon captured exon 2 (270 bp) plus noncoding regions around it. Given this extra data for *MHC-DOB*, we analyzed both the extended *MHC-DOB* sequences and *MHC-DOB* exon 2 for further analyses.

### Defining new alleles

Alleles were defined based on nucleotide sequence variation and amino acid sequence variation. All new *MHC-DRB* and *MHC-DOB* alleles followed the nomenclature proposed by Klein et al. [45] and Van Den Bussche et al. [99, 100]. To define alleles and genotypes within an individual, the merged paired-end sequences were organized from the most frequent to least frequent sequence. Individuals were characterized as homozygotes when the frequency of the second most common sequence was less than 10% relative to the first sequence (90:10; median ratio = 99:1). Individuals were classified as heterozygotes if the ratio of the first to second most frequent sequence was 50:50 to 65:35 (median ratio = 58:42). For *MHC-DRB*, 60 individuals out of the 380 individuals sampled (15.8%) did not classify as a homozygote or heterozygote using these guidelines, thereby we used pedigree and Sanger sequencing data to confirm their genotypes. When creating the pedigree for our population, parentage was assigned with 95% confidence [64]. We compared pedigree data with allele assignments made for the clear homozygotes and heterozygotes and found that 95.2% of the parental assignments were consistent with the *MHC-DRB* allele assignments and 96.1% of the parental assignments were consistent with the *MHC-DOB* allele assignments. These results reflect the 95% reliability threshold used when creating our pedigree. We therefore did not discard the allele assignments that were inconsistent with the pedigree data. We also further explored the other less frequent alleles and found that they were chimeric alleles. We removed any individuals who did not have Sanger or pedigree data for confirmation (*n* = 5), which reduced the sample size for *MHC-DRB* to 375 individuals. For *MHC-DOB*, all 380 individuals sampled were clear homozygotes or heterozygotes (extended sequence and exon 2).

New *MHC-DRB* alleles whose frequencies were less than 0.67% [5/ (2*375)] in the population were not considered true alleles [67] if we could not further validate them using pedigree and Sanger analysis. While 5 *MHC-DRB* alleles were at less than 0.67% in our population, we were able to validate 3 of these 5 alleles (DRB*28, DRB*29, DRB*30). The other two alleles only occurred once in the population, and these individuals were removed from our DRB dataset for further analysis (Table S1), reducing our sample size further to 373 individuals for *MHC-DRB*. The minimum allele frequency threshold for *MHC-DOB* was 0.66% [5/ (2*380)]. While all *MHC-DOB* exon 2 allele frequencies were greater than this minimum, one allele from our extended *MHC-DOB* sequence data was not. However, we were able to validate this allele using Sanger and pedigree data so it was not removed from the dataset.

The pedigree for our population was created by Newbolt et al. [64]. Sanger sequencing [85] was performed for *MHC-DRB* exon 2 via high throughput sequencing (htSEQ) for individuals born prior to 2008 to augment our MiSeq sequencing data for *MHC-DRB*.

To investigate whether all characterized alleles belonged to a single *MHC-DRB* or *MHC-DOB* locus, we mapped *MHC-DRB* exon 2 and *MHC-DOB* exon 2 alleles to the available draft assembly of the white-tailed deer genome (Ovir.te.1; [87]). *MHC-DRB* alleles mapped to two unplaced scaffolds (NW_018337343.1 and NW_018338651.1), and *MHC-DOB* alleles mapped to one unplaced scaffold (NW_018336049.1). This suggests that there may have been a gene duplication event for *MHC-DRB* in white-tailed deer, at least for the exon 2 portion of the gene. However, patterns seen in our data for *MHC-DRB* are more consistent with the presence of one *MHC-DRB* locus. *MHC-DRB* sequences that were slightly less frequent than the assigned *MHC-DRB* alleles for heterozygotes were chimeric sequences of the two most common *MHC-DRB* alleles for that individual, a commonly known PCR artifact [36]. It is unlikely that all of the individuals in our dataset were completely homozygous at both loci and heterozygous for the same *MHC-DRB* alleles at both loci. Since MHC genes are notoriously difficult to assemble, especially highly variable regions such as *MHC-DRB*, it is possible that in the draft assembly the *MHC-DRB* haplotypes were interpreted as different genes and therefore forced onto different scaffolds when assembling this first version of the white-tailed deer genome [23, 55, 102].

### Test for linkage between MHC-DRB and MHC-DOB

Since *MHC-DRB* and *MHC-DOB* may lie on different MHC II subregions in white-tailed deer, as they are separated by an inversion in other ruminants [8, 16, 82], we examined the degree of linkage between *MHC-DRB* and *MHC-DOB* among unrelated individuals in our white-tailed deer population using GenePop (Option 2, sub-option 1). Since the second exons of *MHC-DRB* and *MHC-DOB* are placed on different scaffolds that were not included in the final assembly of the white-tailed deer genome (*MHC-DRB*, NW_018337343.1; *MHC-DOB*, NW_018336049.1; [87]), we were unable to use the available genomic data for addressing this linkage question.

Therefore, to attempt to address this question here, we first assessed linkage disequilibrium (LD) using individuals that are least likely related to one another (individuals born before the fence was constructed in 2007 and that were not offspring from this early group of deer according to pedigree data; *n* = 69). We then included individuals without assigned parents in our pedigree data (these individuals could be related to others in the population, but we are not 95% confident that they are; *n* = 122) to further assess the possibility of LD between *MHC-DRB* exon 2 and *MHC-DOB* exon 2. This method in GenePop [76, 78] tests the null hypothesis that the loci are independent of one another, or in other words, not closely linked [104]. We used the default settings where dememorization = 1000, batches = 100, and iterations per batch = 1000.

### Genetic relationships among alleles

We estimated phylogenetic distances as the number of nucleotide/amino acid differences among alleles and created gene trees for each gene to understand the relationships between *MHC-DRB* and *MHC-DOB*. Nucleotide and protein alignments of *MHC-DRB* and *MHC-DOB* were created via Geneious (v11.1.5). These alignments were then used to construct phylogenetic trees for the nucleotide and amino acid sequences of both *MHC-DRB* and *MHC-DOB* using IQ-TREE (v1.6.9; [65]). IQ-TREE employed maximum likelihood (ML) for tree inference and automatically determined the best-fit model (−m TEST) via ModelFinder [42] using a standard bootstrap of 1000 replicates (−b 1000). ModelFinder identifies the best-fitting model of sequence evolution that ultimately produced our data. Phylogenetic support for tree splits was determined via bootstrap values, where a split with ≥95% support is considered statistically significant [26]. Outgroups included in the *MHC-DRB* trees were moose (*Alces alces*, [56]), roe deer (*Capreolus capreolus*, [57, 75]), and reindeer (*Rangifer tarandus*, [59]) as these are the closest related species to white-tailed deer with *MHC-DRB* data [33, 71]. More distantly related species could result in a long branch, which may dominate the likelihood and therefore interfere with the tree inference. However, due to a lack of *MHC-DOB* data among artiodactyls, outgroups for the *MHC-DOB* trees were more distantly related species including cow (*Bos taurus*, [112]), sheep (*Ovis aries*, [107]), and red deer (*Cervus elaphus*, [7]). The nucleotide tree for *MHC-DOB* used the extended nucleotide sequences (360 bp), while the amino acid tree used translated *MHC-DOB* exon 2 sequences. The final ML trees were rooted using FigTree (v1.4.4). The *MHC-DRB* trees were rooted with the moose outgroup while the *MHC-DOB* trees were rooted with the cow outgroup. A heatmap was added to the trees via FigTree annotation that corresponded to the Odvi-DRB and Odvi-DOB allele frequencies present in our population.

### Population genetic measures and test for selection

Allele and genotype frequencies were generated for each gene via GenePop (v4.2; Option 5, sub-option1; [76, 78]) to examine the distribution of *MHC-DRB* and *MHC-DOB* alleles in our white-tailed deer population. To assess if these allele frequencies were changing over time, Hardy Weinberg Exact Tests were performed on nucleotide data from the adult founding [2003–2007; *n* = 69 (*MHC-DRB*), *n* = 71 (*MHC-DOB* extended sequence), n = 71 (*MHC-DOB* exon 2)] and adult 2016 datasets [*n* = 118 (*MHC-DRB*), *n* = 119 (*MHC-DOB* extended sequence), n = 119 (*MHC-DOB* exon 2)] with GenePop (Option 1) using the probability-test (sub-option 3; [35, 37]). We also checked for deviations from Hardy Weinberg Equilibrium using score tests [79] to evaluate the presence of heterozygote excess (sub-option 2) or deficiency (sub-option 1) in our founding and 2016 populations. Score tests are more powerful tests than the probability test [79], which will further aid in the detection of deviations from HWE. However, since our population sizes are relatively small, we cannot rule out the effect of drift on these tests. All tests employed default settings (dememorization number = 1000, number of batches = 100, number of iterations per batch = 1000).

Nucleotide (π) and haplotype (Hd) diversity [63] were calculated for both *MHC-DRB* exon 2 and *MHC-DOB* (extended sequence and exon 2) using DnaSP v5.10.00 [80] to assess the genetic diversity present within our population. We also performed several neutrality tests (Tajima’s D, Fu’s Fs, Fu and Li’s F*, Fu and Li’s D*) in DnaSP to examine possible evidence of selection or drift at these loci in our white-tailed deer population. These tests were done using the founding, unrelated population (as described above) and the adults present in the population in 2016 (as described above). Pairwise sequence divergence for nucleotide and amino acid sequences were calculated using MEGA X (v10.0.5; [47]), using maximum composite likelihood for nucleotide distances and a Poisson correction for amino acid distances.

Lastly, to test for evidence of positive selection, SNAP (Synonymous Non-synonymous Analysis Program; v2.1.1; [46]) was used to calculate synonymous and non-synonymous substitution rates and the ratio dN/dS for *MHC-DRB* exon 2 and *MHC-DOB* exon 2.

## Supplementary information

**Additional file 1:Table S1.***MHC-DRB* exon 2 alleles whose frequencies did not meet the minimum allele frequency (0.67%). No pedigree or sanger data was available to validate these sequences, and they only occurred once in our white-tailed deer population. **Table S2.***MHC-DRB* exon 2 alleles found in our white-tailed deer population and their frequencies. (^ indicates that these alleles translated into the same amino acid sequence). **Table S3.** number of nucleotide (below diagonal) and amino acid (above diagonal) differences between *MHC-DRB* exon 2 alleles for white-tailed deer. **Table S4.** Genotype frequencies (%) for *MHC-DRB* exon 2 alleles in our white-tailed deer population. **Table S5.***MHC-DOB* alleles for both the extended sequence (360 bp) and exon 2 (270 bp) found in our white-tailed deer population and their frequencies. (^#^ and ^ indicates that these alleles translated into the same amino acid sequence). **Table S6.** Number of nucleotide (below diagonal) and amino acid (above diagonal) differences between the extended *MHC-DOB* sequence (360 bp) alleles for white-tailed deer. The amino acid differences correspond to the amino acid differences seen in *MHC-DOB* exon 2 (table S7). **Table S7.** Number of nucleotide (below diagonal) and amino acid (above diagonal) differences between *MHC-DOB* exon 2 (270 bp) alleles for white-tailed deer. **Table S8.** Genotype frequencies (%) for the extended *MHC-DOB* sequence (360 bp) alleles in our white-tailed deer population. **Table S9.** Genotype frequencies (%) for *MHC-DOB* exon 2 (270 bp) alleles in our white-tailed deer population. **Figure S1.** Cumulative mean codon-by-codon ratio of synonymous to nonsynonymous substitutions (dS/dN) for *MHC-DRB* exon 2. Nonsynonymous substitutions are significantly more common than synonymous substitutions for *MHC-DRB* exon 2 in white-tailed deer. **Figure S2.** Cumulative mean codon-by-codon ratio of synonymous to non-synonymous substitutions (dS/dN) for *MHC-DOB* exon 2. Synonymous substitutions are overall more common than nonsynonymous substitutions

## Data Availability

All raw sequence data is available on the NCBI Sequence Read Archive (SRA accession # PRJNA533917). The 12 new *MHC-DRB* exon 2 alleles and the 11 extended *MHC-DOB* sequences have been deposited in Genbank under accession numbers MK952679- MK952690 and MK952691- MK952701, respectively.

## Abbreviation

MHC: Major histocompatibility complex

## References

[1] Acevedo-Whitehouse K, Gulland FMD, Bowen L (2018). MHC class II DRB diversity predicts antigen recognition and is associated with disease severity in California sea lions naturally infected with *Leptospira interrogans*. Infect Genet Evol.

[2] Allen M, Kalantari M, Ylitalo N, Pettersson B, Hagmar B, Scheibenpflug L, Johansson B, Petterson U, Gyllensten U (1996). HLA DQ-DR haplotype and susceptibility to cervical carcinoma: indications of increased risk for development of cervical carcinoma in individuals infected with HPV 18. Tissue Antigens..

[3] Allen RH (1965). History and results of deer restocking in Alabama.

[4] Amills M, Ramiya V, Norimine J, Lewin HA (1998). The major histocompatibility complex of ruminants. Rev Sci Tech Off Int Epiz..

[5] Andersson L, Gustafsson K, Jonsson AK, Rask L (1991). Concerted evolution in a segment of the first domain exon of polymorphic MHC class II β loci. Immunogenetics..

[6] Andersson L, Lunden A, Sigurdardottir S, Davies CJ, Rask L (1988). Linkage relationships in the bovine MHC region. High recombination frequency between class II subregions. Immunogenetics..

[7] Bana NA, Nagy T, Nyiri A, Nagy J, Frank K, Horn P, Steger V, Orosz L, Barta E (2016). Genome sequencing and assembly of Hungarian red deer, *Cervus elaphus hippelaphus*.

[8] Band M, Larson JH, Womack JE, Lewin HA (1998). A radiation hybrid map of BTA23: identification of a chromosomal rearrangement leading to separation of the cattle MHC class II subregions. Genomics..

[9] Behl JD, Verma NK, Behl R, Mukesh M, Ahlawat SPS (2007). Characterization of genetic polymorphism of the bovine lymphocyte antigen DRB3.2 locus in Kankrej cattle (*Bos indicus*). J Dairy Sci.

[10] Bernatchez L, Landry C (2003). MHC studies in nonmodel vertebrates: what have we learned about natural selection in 15 years?. J Evol Biol.

[11] Biswas S, Akey JM (2006). Genomic insights into positive selection. Trends Genet.

[12] Bolger AM, Lohse M, Usadel B (2014). Trimmomatic: a flexible trimmer for Illumina sequence data. Bioinformatics..

[13] Borghans JAM, De Boer RJ, Segel LA, Cohen IR (2000). Diversity in the immune system. Design principles for immune system & other distributed autonomous systems.

[14] Chennagiri N, White EJ, Frieden A, Lopez E, Lieber DS, Nikiforov A, Ross T, Batorsky R, Hansen S, Lip V, Luquette LJ, Maucheli E, Margulies D, Milos PM, Napolitano N, Nizzari MM, Yu T, Thompson JF (2016). Orthogonal NGS for high throughput clinical diagnostics. Sci Rep..

[15] Chiang JPW, Lamey T, McLaren T, Thompson JA, Montgomery H, Roach D (2015). Progress and prospects of next-generation sequencing testing for inherited retinal dystrophy. Expert Rev Mol Diagn.

[16] Childers CP, Newkirk HL, Honeycutt DA, Ramlachan N, Muzney DM, Sodergren E, Gibbs RA, Weinstock GM, Womack JE, Skow LC (2005). Comparative analysis of the bovine MHC class IIb sequence identifies inversion breakpoints and three unexpected genes. Anim Genet.

[17] Cicconardi F, Marcatili P, Arthofer W, Schlick-Steiner BC, Steiner FM (2017). Positive diversifying selection is a pervasive adaptive force throughout the Drosophila radiation. Mol Phylogenet Evol.

[18] Coffroth MA, Lasker HR, Diamond ME, Bruenn JA, Bermingham EJMB (1992). DNA fingerprints of a gorgonian coral: a method for detecting clonal structure in a vegetative species. Mar Biol.

[19] Couvillion CE, Nettles VF, Rawlings CA, Joyner RL (1986). Elaeophorosis in white-tailed deer: pathology of the natural disease and its relation to oral food impactions. J Wildl Dis.

[20] De S, Singh RK, Butchaiah G (2002). MHC-DRB exon 2 allele polymorphism in Indian river buffalo (*Bubalus bubalis*). Anim Genet.

[21] De S, Singh RK, Brahma B (2011). Allelic diversity of major histocompatibility complex class II DRB gene in Indian cattle and buffalo. Mol Biol Int.

[22] De Vries RRP, Mehra NK, Vaidya MC, Gupte MD, Khan PM, van Rood JJ (1980). HLA-linked control of susceptibility to tuberculoid leprosy and association with HLA-DR types. Tissue Antigens..

[23] Dilthey A, Cox C, Iqbal Z, Nelson MR, McVean G (2015). Improved genome inference in the MHC using a population reference graph. Nat Genet..

[24] Ditchkoff SS, Hoofer SR, Lochmiller RL, Masters RE, Van Den Bussche RA (2005). MHC-DRB evolution provides insight into parasite resistance in white-tailed deer. Southwest Nat.

[25] Ellsworth DL, Honeycutt RL, Silvy NJ, Bickham JW, Klimstra WD (1994). White-tailed deer restoration to the southeastern United States: evaluating mitochondrial DNA and allozyme variation. J Wildl Manag..

[26] Felsenstein J (1985). Confidence limits on phylogenies: an approach using the bootstrap. Evolution.

[27] Fernandez-de-Mera IG, Vicente J, Naranjo V, Fierro Y, Garde JJ, de la Fuente J, Gortazar C (2009). Impact of major histocompatibility complex class II polymorphisms on Iberian red deer parasitism and life history traits. Infect Genet Evol.

[28] Fletch AL, Karstad LH (1971). Studies on the pathogenesis of experimental epizootic hemorrhagic disease of white-tailed deer. Can J Com Med.

[29] Gao J, Liu K, Liu H, Blair HT, Li G, Chen C, Tan P, Ma RZ (2010). A complete DNA sequence map of the ovine major histocompatibility complex. BMC Genomics.

[30] Gaur LK, Nepom GT (1996). Ancestral major histocompatibility complex DRB genes beget conserved patterns of localized polymorphisms. Proc Natl Acad Sci USA.

[31] Genome Reference Consortium (2019). Genome Reference Consortium Human Build 38 patch release 13 (GRCh38.p13), 02/28/2019.

[32] Ghodke Y, Joshi K, Chopra A, Patwardhan B (2005). HLA and disease (review). Eur J Epidemiol..

[33] Gilbert C, Ropiquet A, Hassanin A (2006). Mitochondrial and nuclear phylogenies of Cervidae (Mammalia, Ruminantia): Systematics, morphology, and biogeography. Mol Phylogenet Evol.

[34] Godot V, Harraga S, Beurton I, Tiberghien P, Sarciron E, Gottstein B, Vuitton DA (2000). Resistance/susceptibility to *Echinococcus multilocularis* infection and cytokine profile in humans. II. Influence of the HLA B8, DR3, DQ2 haplotype. Clin Exp Immunol.

[35] Guo SW, Thompson EA. Performing the Exact Test of Hardy-Weinberg Proportion for Multiple Alleles. Biometrics. 1992;48(2):361–72. 1637966

[36] Haas BJ, Gevers D, Earl AM, Feldgarden M, Ward DV, Giannoukos G, Ciulla D, Tabbaa D, Highlander SK, Sodergren E, Methe B, DeSantis TZ, Petrosino JF, Knight R, Birren BW. Chimeric 16S rRNA sequence formation and detection in Sanger and 454-pyrosequenced PCR amplicons. Genome Res. 2011;21(3):494–504. 10.1101/gr.112730.110 PMC3044863 21212162

[37] Haldane JBS. An exact test for randomness of mating. J Genet. 1954;52(3):631–5.

[38] Hedrick PW (1994). Evolutionary genetics of the major histocompatibility complex. Am Nat.

[39] Hewitt DG (2011). Biology and management of white-tailed deer.

[40] Janeway CA, Travers P, Walport M (2001). Immunobiology: the immune system in health and disease.

[41] Jordanova A, Kalaydjieva L, Savov A, Claustres M, Schwarz M, Estivill X, Angelicheva D, Haworth A, Casals T, Kremensky I (1997). SSCP analysis: a blind sensitivity trial. Hum Mutat.

[42] Kalyaanamoorthy S, Minh BQ, Wong TKF, von Haeseler A, Jermijn LS (2017). ModelFinder: Fast model selection for accurate phylogenetic estimates. Nature Methods.

[43] Kamiya T, O’Dwyer K, Westerdahl H, Senior A, Nakagawa S (2014). A quantitative review of MHC-based mating preference: the role of diversity and dissimilarity. Mol Ecol.

[44] Kilpatrick HJ, DeNicola AJ, Ellingwood MR (1996). Comparison of standard and transmitter-equipped darts for capturing white-tailed deer. Wildl Soc Bull.

[45] Klein J, Bontrop RE, Dawkins RL, Erlich HA, Hyllensten UB, Heise ER, Jones PP, Parham P, Wakeland EK, Watkins DI (1990). Nomenclature for the major histocompatibility complexes of different species: a proposal. Immunogenetics.

[46] Korber B, Rodrigo AG, Learn GH (2000). HIV Signature and Sequence Variation Analysis. Computational Analysis of HIV Molecular Sequences.

[47] Kumar S, Stecher G, Li M, Knyaz C, Tamura K (2018). MEGA X: Molecular evolutionary genetics analysis across computing platforms. Mol Biol Evol.

[48] Larruskain A, Minguijon E, Garcia-Etxebarria K, Moreno B, Arostegui I, Juste RA, Jugo BM (2010). MHC class II DRB1 gene polymorphism in the pathogenesis of Maedi-Visna and pulmonary adenocarcinoma viral diseases in sheep. Immunogenetics..

[49] Leberg PL, Stangel PW, Hillestad HO, Marchinton R, Smith MH (1994). Genetic structure of reintroduced wild turkey and white-tailed deer populations. J Wildl Manag..

[50] Li L, Wang BB, Ge YF, Wan QH (2014). Major histocompatibility complex class II polymorphisms in forest musk deer (*Moschus berezovskii*) and their probable associations with purulent disease. Int J Immunogenet..

[51] Liu HY (2004). The diagnosis and treatment of the purulent disease of musk deer. J Southwest Uni Sci Tech..

[52] McDonald JS, Miller KV (1993). A history of white-tailed deer restocking in the United States 1878 to 1992.

[53] McDonald JS, Miller KV (2004). A history of white-tailed deer restocking in the United States 1878 to 2004.

[54] Mellins ED, Stern LJ (2014). HLA-DM and HLA-DO, key regulators of MHC-II processing and presentation. Curr Opin Immunol.

[55] Migalska M, Sebastian A, Konczal M, Kotlík P, Radwan J (2016). De novo transcriptome assembly facilitates characterization of fast-evolving gene families, MHC class I in the bank vole (*Myodes glareolus*). Heredity.

[56] Mikko S, Andersson L (1995). Low major histocompatibility complex class II diversity in European and North American moose. Proc Natl Acad Sci USA..

[57] Mikko S, Lewin HA, Andersson L (1997). A phylogenetic analysis of cattle DRB3 alleles with a deletion of codon 65. Immunogenetics.

[58] Mikko S, Spencer M, Morris B, Stabile S, Basu T, Stormont C, Andersson L (1997). A comparative analysis of Mhc DRB3 polymorphism in the American bison (*Bison bison*). J Hered..

[59] Mikko S, Roed K, Schmutz S, Andersson L (1999). Monomorphism and polymorphism at the Mhc DRB loci in domestic and wild ruminants. Immunol Rev.

[60] Miller BF, Muller LI, Storms TN, Ramsay EC, Osborn DA, Warren RJ, Miller KV, Adams KA (2003). A comparison of carfentanil/xylazine and Telazol/xylazine for immobilization of white-tailed deer. J Wildl Dis..

[61] Miller BF, Muller LI, Doherty T, Osborn DA, Miller KV, Warren RJ (2004). Effectiveness of antagonists for tiletamine-zolazepam/xylazine immobilization in female white-tailed deer. J Wildl Dis..

[62] NCBI: National Center for Biotechnology Information. HLA-DOB major histocompatibility complex, class II, DO beta [*Homo sapiens* (human)]. Gene. 2019;3112 Available at: https://www.ncbi.nlm.nih.gov/gene/3112#gene-expression. Accessed 18 Mar 2019.

[63] Nei M (1987). Molecular evolutionary genetics.

[64] Newbolt CH, Acker PK, Neuman TJ, Hoffman SI, Ditchkoff SS, Steury TD (2017). Factors influencing reproductive success in male white-tailed deer. J Wildl Manag.

[65] Nguyen LT, Schmidt HA, von Haeseler A, Minh BQ (2015). IQ-TREE: A fast and effective stochastic algorithm for estimating maximum likelihood phylogenies. Mol Biol Evol.

[66] Nieto A, Beraun Y, Callado MD, Caballero A, Alonso A, Gonzalez A, Martin J (2000). HLA haplotypes are associated with differential susceptibility to *Trypanosoma cruzi* infection. Tissue Antigens.

[67] NRC: National Research Council (1996). The evaluation of forensic DNA evidence.

[68] Park C, Russ I, Da Y, Lewin HA (1995). Genetic mapping of F13A to BTA23 by sperm typing: difference in recombination rate between bulls in the DYA-PRL interval. Genomics.

[69] Park C, Frank MT, Lewin HA (1999). Fine-mapping of a region of variation in recombination rate on BTA23 to the D23S22-D23S23 interval using sperm typing and meiotic breakpoint analysis. Genomics..

[70] Park YH, Joo YS, Park JY, Moon JS, Kim SH, Kwon NH, Ahn JS, Davis WC, Davies CJ (2004). Characterization of lymphocyte subpopulations and major histocompatibility complex haplotypes of mastitis-resistant and susceptible cows. J Vet Sci.

[71] Pitra C, Fickel J, Meijaard E, Groves C (2004). Evolution and phylogeny of old world deer. Mol Phylogenetics Evol..

[72] Poluektov YO, Kim A, Sadegh-Nasseri S (2013). HLA-DO and its role in MHC class II antigen presentation. Front Immunol..

[73] Prestwood AK, Hayes FA, Eve JH, Smith JF (1973). Abomasal helminths of white-tailed deer in southeastern United States, Texas, and the Virgin Islands. J Am Vet Med A..

[74] Prestwood AK, Kellogg FE (1971). Naturally occurring Haemonchosis in a white-tailed deer. J Wildl Dis..

[75] Quemere E, Galan M, Cosson JF, Klein F, Aulagnier S, Gilot-Fromont E, Merlet J, Bonhomme M, Hewison AJM, Charbonnel N (2015). Immunogenetic heterogeneity in a widespread ungulate: the European roe deer (*Capreolus capreolus*). Mol Ecol..

[76] Raymond M, Rousset F (1995). GENEPOP (version 1.2): population genetics software for exact tests and ecumenicism. J Hered..

[77] Renard C, Hart E, Sehra H, Beasley H, Coggill P, Howe K, Harrow J, Gilbert J, Sims S, Rogers J, Ando A, Shigenari A, Shiina T, Inoko H, Chardon P, Beck S (2006). The genomic sequence and analysis of the swine major histocompatibility complex. Genomics..

[78] Rousset F (2008). Genepop’007: a complete reimplementation of the Genepop software for Windows and Linux. Mol Ecol Resources.

[79] Rousset F, Raymond M (1995). Testing heterozygote excess and deficiency. Genetics.

[80] Rozas J, Sanchez-DelBarrio JC, Messeguer X, Rozas R (2003). DnaSP, DNA polymorphism analyses by the coalescent and other methods. Bioinformatics..

[81] Rozas J (2009). DNA sequence polymorphism analysis using DnaSP. Methods Mol Biol.

[82] Rozen BD, Bickhart DM, Koren S, Schnabel RD, Hall R, Zimin A, Dreischer C, Schultheiss S, Schroeder SG, Elsik CG, Couldrey C, Liu GE, Phillippy AM, Van Tassell CP, Smith TPL, Medrano JF (2017). ARS-UCD1.2, Direct Submission, 06/29/2017.

[83] Ruan R, Ruan J, Wan XL, Zheng Y, Chen MM, Zheng JS, Wang D (2016). Organization and characteristics of the major histocompatibility complex class II region in the Yangtze finless porpoise (*Neophocaena asiaeorientalis asiaeorientalis*). Sci Rep.

[84] Sa ALA, Breaux B, Burlamaqui TCT, Deiss TC, Sena L, Criscitiello MF, Schneider MPC (2019). The marine mammal class II major histocompatibility complex organization. Front Immunol..

[85] Sanger F, Coulson AR (1975). A rapid method for determining sequences in DNA by primed synthesis with DNA polymerase. J Mol Biol..

[86] Schook LB, Lamont SJ (1996). The major histocompatibility complex region of domestic animal species.

[87] Seabury CM, Bhattarai EK, Taylor JF, Viswanathan GG, Cooper SM, Davis DS, Dowd SE, Lockwood ML, Seabury PM (2011). Genome-wide polymorphism and comparative analyses in the white-tailed deer (*Odocoileus virginianus*): a model for conservation genomics. PLoS One.

[88] Seddon JM, Ellegren H (2002). MHC class II genes in European wolves: a comparison with dogs. Immunogenetics..

[89] Severinghaus CW (1949). Tooth development and wear as criteria of age in white-tailed deer. J Wildl Manag..

[90] Sigurdardottir S, Borsch C, Gustaffson K, Andersson L (1991). Cloning and sequence analysis of 14 DRB alleles of the bovine major histocompatibility complex by using the polymerase chain reaction. Anim Genet..

[91] Sikes RS, Gannon WL (2011). Guidelines of the American Society of Mammalogists for the use of wild mammals in research. J Mammal.

[92] Singh PK, Singh SV, Singh MK, Saxena VK, Horin P, Singh AV, Sohal JS (2012). Effect of genetic variation in the MHC Class II DRB region on resistance and susceptibility to Johne’s disease in endangered Indian Jamunapari goats. Int J Immunogenet..

[93] Sommer S (2005). The importance of immune gene variability (MHC) in evolutionary ecology and conservation. Front Zool.

[94] Sommer S, Courtiol A, Mazzoni C (2013). MHC genotyping of non-model organisms using next-generation sequencing: a new methodology to deal with artefacts and allelic dropout. BMC Genomics..

[95] Steinmetz M, Stephan D, Fischer-Lindahl K (1986). Gene organization and recombination hotspots in the murine major histocompatibility complex. Cell..

[96] Stone RT, Muggli-Cockett NE (1993). BoLA-DIB: species distribution, linkage with DOB, and Northern analysis. Anim Genet..

[97] Swarbrick PA, Schwaiger FW, Epplen JT, Buchan GS, Griffin JFT, Crawford AM (1995). Cloning and sequencing of expressed DRB genes of the red deer (*Cervus elaphus*) Mhc. Immunogenetics..

[98] Termijtelen A, Meera Khan P, Shaw S, van Rood JJ (1983). Mapping SB in relation to HLA and GLO1 using cells from first-cousin marriage offspring. Immunogenetics..

[99] Van Den Bussche RA, Hoofer SR, Lochmiller RL (1999). Characterization of Mhc-DRB allelic diversity in white-tailed deer (*Odocoileus virginianus*) provides insight into *Mhc*-*DRB* allelic evolution within Cervidae. Immunogenetics..

[100] Van Den Bussche RA, Ross TG, Hoofer SR (2002). Genetic variation at a major histocompatibility locus within and among populations of white-tailed deer (*Odocoileus virginianus*). J Mammal.

[101] Van Eijk MJT, Beever JE, Da Y, Stewart JA, Nicholaides GE, Green CA, Lewin HA (1995). Genetic mapping of BoLa-A, CYP21, DRB3, DYA and PRL on BTA23. Mamm Genome.

[102] Viļuma A, Mikko S, Hahn D, Skow L, Andersson G, Bergström TF (2017). Genomic structure of the horse major histocompatibility complex class II region resolved using PacBio long-read sequencing technology. Sci Rep.

[103] Wan QH, Zeng CJ, Ni XW, Pan HJ, Fang SG (2009). Giant panda genomic data provide insight into the birth-and-death process of mammalian major histocompatibility complex class II genes. PLoS One.

[104] Weir BS (1996). Genetic Data Analysis II.

[105] Wieczorek M, Abualrous ET, Sticht J, Alvaro-Benito M, Stolzenberg S, Noe F, Freund C (2017). Major histocompatibility complex (MHC) class I and MHC class II proteins: conformational plasticity in antigen presentation. Front Immunol.

[106] Wright S (1938). Size of population and breeding structure in relation to evolution. Science.

[107] Wright H, Redmond J, Ballingall KT (1996). The sheep orthologue of the HLA-DOB gene. Immunogenetics..

[108] Yang Z, Nielsen R, Goldman N, Pedersen AMK (2000). Codon-substitution models for heterogeneous selection pressure at amino acid sites. Genetics..

[109] Zhang J, Kobert K, Flouri T, Stamatakis A (2014). PEAR: a fast and accurate Illumina Paired-End reAd mergeR. Bioinformatics..

[110] Zhang Z, Sun X, Chen M, Li L, Ren W, Xu S, Yang G. Genomic organization and phylogeny of MHC class II loci in cetaceans. J Hered. 2019;esz005. 10.1093/jhered/esz005 Accessed 11 Mar 2020. 10.1093/jhered/esz005 30844043

[111] Zhou X, Xu S, Yang Y, Zhou K, Yang G (2011). Phylogenomic analyses and improved resolution of Cetartiodactyla. Mol Phylogenet Evol..

[112] Zimin AV, Delcher AL, Florea L, Kelley DR, Schatz MC, Puiu D, Hanrahan F, Pertea G, Van Tassell CP, Sonstegard TS, Marcais G, Roberts M, Subramanian P, Yorke JA, Salzberg SL (2009). A whole-genome assembly of the domestic cow, *Bos taurus*. Genome Biol.

