# Supplementary material for: Characterization of two MHC II genes (DOB, DRB) in white-tailed deer (Odocoileus virginianus)
Source: BMC Genet. 2020 Jul 29;21:83. doi: 10.1186/s12863-020-00889-5 (PMC7392685; doi:10.1186/s12863-020-00889-5)
Supplement: Supplementary file 1 — Additional file 1:Table S1.MHC-DRB exon 2 alleles whose frequencies did not meet the minimum allele frequency (0.67%). No pedigree or sanger data was available to validate these sequences, and they only occurred once in our white-tailed deer population. Table S2.MHC-DRB exon 2 alleles found in our white-tailed deer population and their frequencies. (^ indicates that these alleles translated into the same amino acid sequence). Table S3. number of nucleotide (below diagonal) and amino acid (above diagonal) differences between MHC-DRB exon 2 alleles for white-tailed deer. Table S4. Genotype frequencies (%) for MHC-DRB exon 2 alleles in our white-tailed deer population. Table S5.MHC-DOB alleles for both the extended sequence (360 bp) and exon 2 (270 bp) found in our white-tailed deer population and their frequencies. (# and ^ indicates that these alleles translated into the same amino acid sequence). Table S6. Number of nucleotide (below diagonal) and amino acid (above diagonal) differences between the extended MHC-DOB sequence (360 bp) alleles for white-tailed deer. The amino acid differences correspond to the amino acid differences seen in MHC-DOB exon 2 (table S7). Table S7. Number of nucleotide (below diagonal) and amino acid (above diagonal) differences between MHC-DOB exon 2 (270 bp) alleles for white-tailed deer. Table S8. Genotype frequencies (%) for the extended MHC-DOB sequence (360 bp) alleles in our white-tailed deer population. Table S9. Genotype frequencies (%) for MHC-DOB exon 2 (270 bp) alleles in our white-tailed deer population. Figure S1. Cumulative mean codon-by-codon ratio of synonymous to nonsynonymous substitutions (dS/dN) for MHC-DRB exon 2. Nonsynonymous substitutions are significantly more common than synonymous substitutions for MHC-DRB exon 2 in white-tailed deer. Figure S2. Cumulative mean codon-by-codon ratio of synonymous to non-synonymous substitutions (dS/dN) for MHC-DOB exon 2. Synonymous substitutions are overall more common than nonsy [file 12863_2020_889_MOESM1_ESM.pdf]

Characterization of two MHC II genes (DOB, DRB) in white-tailed deer (*Odocoileus virginianus*)

Immunogenetics

Natascha M.D. Ivy-Israel[a], Carolyn E. Moore[a], Tonia S. Schwartz[b], Stephen S. Ditchkoff[a]

[a] School of Forestry and Wildlife Sciences, Auburn University, AL 36849, USA

[b] Department of Biological Sciences, Auburn University, AL 36849, USA

[c] Corresponding Author: Natascha Ivy-Israel, nmi0001@auburn.edu, 469-396-6451

TSS: ORCID: 0000-0002-7712-2810

Table S1. *MHC-DRB* exon 2 alleles whose frequencies did not meet the minimum allele frequency (0.67%). No pedigree or Sanger data was available to validate these sequences, and they only occurred once in our white-tailed deer population

>Odvi-DRB*31 [organism=Odocoileus virginianus]

GGAGTATCATAAGGCCGAGTGTCATTTCTCCAACGGGACGCAGCGGGTGCGGTTCCTGGACAGATACA
TCTATAACCAGGAAGAGTACGTGCGCTTCGACAGCGACGTGGGCGAGTACCGGGCGGTGACAGAGCT
GGGGCGGCCGGACGCCGAGGACTGGAACAGCCGGAAGGAGCTCCTGGAGCAGAGGCGGGCCGAGGT
GGACACGTACTGCAGACACAACTACGGGGTTATTGAGAGTTTCACTGTG

>Odvi-DRB*32 [organism=Odocoileus virginianus]

GGAGCATCATAAGGCCGAGTGTCATTTCTCCAACGGGACGCAGGGGGTGCAGTTCCTGCAGAGATACG
TCTATAACCAGGAAGAGTACGTGCGCTTCGACAGCAACGTGGGCGAGTACCGAGCGGTGACCGAGCT
GGGGCGGACGGACGCCAAGTACTATAACAGCCAGAAGGAGTTACTGGAGCAGAAGCGGGCCTCGGTG
GACACGTACTGCAGACACAACTACGGGGTCGGTGAGAGTTTCACTGTG

Table S2. *MHC-DRB* exon 2 alleles found in our white-tailed deer population and their frequencies. (^ indicates that these alleles translated into the same amino acid sequence)

	Frequency (%)	
Allele	Nucleotide	Amino Acid
Odvi-DRB*01	7.37	7.37
Odvi-DRB*05	0.80	0.80
Odvi-DRB*06	0.94	11.66^
Odvi-DRB*10	22.39	22.39
Odvi-DRB*12	2.95	2.95
Odvi-DRB*14	12.47	12.47
Odvi-DRB*16	7.64	7.64
Odvi-DRB*19	10.72	11.66^
Odvi-DRB*20	14.61	14.61
Odvi-DRB*21	2.55	2.55
Odvi-DRB*22	2.55	2.55
Odvi-DRB*23	3.35	3.35
Odvi-DRB*24	2.28	2.28
Odvi-DRB*25	6.03	6.03
Odvi-DRB*26	1.07	1.07
Odvi-DRB*27	0.80	0.80
Odvi-DRB*28	0.40	0.40
Odvi-DRB*29	0.54	0.54
Odvi-DRB*30	0.54	0.54

Table S3. Number of nucleotide (below diagonal) and amino acid (above diagonal) differences between *MHC-DRB* exon 2 alleles for white-tailed deer.

	DRB*01	DRB*02	DRB*03	DRB*04	DRB*05	DRB*06	DRB*07	DRB*08	DRB*09	DRB*10	DRB*11	DRB*12	DRB*13	DRB*14	DRB*15	DRB*16	DRB*17	DRB*18	DRB*19	DRB*20	DRB*21	DRB*22	DRB*23	DRB*24	DRB*25	DRB*26	DRB*27	DRB*28	DRB*29	DRB*30
DRB*01	-	20	12	8	11	21	21	9	3	11	20	12	12	13	15	16	9	12	21	12	14	15	10	11	13	8	23	9	8	17
DRB*02	31	-	24	21	18	23	5	18	17	18	21	21	25	26	23	18	20	23	23	25	23	20	19	23	24	21	7	21	22	25
DRB*03	19	33	-	15	18	25	24	17	10	16	22	22	7	13	11	25	10	21	25	14	14	22	12	17	15	12	26	14	13	8
DRB*04	16	35	23	-	18	23	19	13	10	14	18	16	13	13	10	20	10	15	23	12	17	15	6	8	14	8	21	7	8	17
DRB*05	17	26	23	28	-	20	19	10	8	4	18	12	20	24	22	21	17	13	20	23	15	11	16	21	16	18	21	19	19	22
DRB*06	33	39	42	40	30	-	24	20	20	20	26	22	25	27	26	27	22	21	0	27	23	21	23	25	21	22	25	23	23	27
DRB*07	32	8	34	30	26	39	-	18	18	16	19	22	24	25	22	19	19	24	24	24	23	20	17	21	24	20	2	19	20	24
DRB*08	14	25	22	22	11	30	26	-	9	10	17	10	16	20	19	17	13	14	20	19	15	12	13	17	14	15	20	15	13	20
DRB*09	4	27	15	20	13	34	28	12	-	8	22	14	12	16	14	17	9	15	20	15	17	15	8	13	16	10	20	11	11	15
DRB*10	21	31	22	22	8	34	24	15	17	-	17	14	17	21	19	23	14	15	20	20	13	13	12	17	15	15	18	15	15	19
DRB*11	28	33	31	29	24	38	31	25	31	27	-	19	20	20	17	23	23	21	26	19	15	17	20	22	15	22	21	21	21	22
DRB*12	18	31	31	25	15	32	33	15	22	21	28	-	18	17	22	17	14	11	22	16	11	11	19	14	14	12	24	12	15	22
DRB*13	18	35	9	24	24	42	35	21	16	25	30	26	-	8	10	22	7	17	25	9	11	20	12	12	14	9	26	9	13	5
DRB*14	17	37	19	22	31	45	38	28	21	32	28	24	14	-	12	24	11	15	27	1	11	20	15	5	12	10	27	9	11	12
DRB*15	21	33	14	15	27	45	34	24	19	26	26	29	15	18	-	23	15	21	26	11	16	21	10	16	15	16	24	15	17	12
DRB*16	25	25	36	32	31	41	28	26	26	37	33	29	33	35	34	-	19	21	27	23	24	20	21	21	24	19	21	19	19	26
DRB*17	17	30	13	18	23	40	27	20	15	19	34	23	10	18	21	32	-	12	22	12	12	16	9	7	16	2	21	4	8	12
DRB*18	18	34	29	24	16	33	36	19	22	22	29	13	24	21	27	33	21	-	21	15	10	12	18	13	8	11	26	11	14	21
DRB*19	34	40	43	41	31	1	40	31	35	35	39	33	43	46	46	42	41	34	-	27	23	21	23	25	21	22	25	23	23	27
DRB*20	15	35	21	20	29	43	36	26	19	30	26	22	16	2	16	33	20	21	44	-	10	19	14	6	11	11	26	10	12	13
DRB*21	19	32	20	26	17	34	33	20	23	18	21	14	17	17	22	36	19	13	35	15	-	16	19	16	6	11	25	13	16	16
DRB*22	20	28	28	24	13	31	28	15	20	19	25	13	27	28	28	29	24	16	32	26	19	-	17	16	14	16	22	15	14	24
DRB*23	21	31	21	15	25	44	27	22	17	21	32	30	21	23	17	36	15	29	45	21	30	28	-	11	18	11	19	10	11	14
DRB*24	17	37	25	15	31	44	33	27	21	27	32	22	20	7	24	34	12	21	45	9	24	26	17	-	17	5	23	4	8	16
DRB*25	18	33	23	21	18	31	33	17	22	21	21	17	22	19	21	33	26	10	32	17	9	16	29	26	-	15	26	15	14	18
DRB*26	14	33	17	14	26	38	30	24	18	22	32	19	14	15	23	32	4	18	39	17	16	24	19	8	23	-	22	2	7	14
DRB*27	36	12	38	34	30	41	4	30	32	28	35	37	39	42	38	32	21	40	42	40	37	32	31	37	37	34	-	21	22	26
DRB*28	14	34	19	14	26	38	29	24	18	22	32	19	14	15	23	32	6	18	39	17	18	24	18	8	23	2	33	-	6	12
DRB*29	14	34	20	14	27	39	29	22	18	23	33	24	21	17	26	27	13	22	40	19	24	22	21	12	21	10	33	10	-	15
DRB*30	22	34	9	28	25	43	34	25	18	26	32	30	6	18	17	37	14	28	44	20	21	31	22	24	26	18	38	16	23	-

Table S4. Genotype frequencies (%) for *MHC-DRB* exon 2 alleles in our white-tailed deer population.

	DRB*01	DRB*05	DRB*06	DRB*10	DRB*12	DRB*14	DRB*16	DRB*19	DRB*20	DRB*21	DRB*22	DRB*23	DRB*24	DRB*25	DRB*26	DRB*27	DRB*28	DRB*29	DRB*30
DRB*01	0.80																		
DRB*05	0	0																	
DRB*06	0.27	0	0																
DRB*10	2.95	0.80	0.54	4.83															
DRB*12	0.54	0	0	1.88	0														
DRB*14	2.15	0	0	4.56	0.27	2.41													
DRB*16	1.07	0	0	3.75	0	1.07	0.27												
DRB*19	1.34	0	0	3.49	0.80	3.49	2.41	0.80											
DRB*20	1.07	0.54	0.54	6.97	1.61	3.49	2.68	2.41	2.15										
DRB*21	0.54	0	0.27	1.07	0	0.80	0.80	1.07	0.54	0									
DRB*22	0.27	0	0	1.07	0.27	0	0.80	0.80	1.07	0.54	0								
DRB*23	0.80	0.27	0	1.61	0	1.07	0.27	0.54	1.07	0	0.54	0							
DRB*24	0.80	0	0	1.07	0	1.07	0	0.80	0.27	0	0	0.27	0						
DRB*25	1.07	0	0.27	3.21	0.54	0.80	0.80	1.88	2.15	0	0.54	0.27	0.27	0					
DRB*26	0	0	0	1.07	0	0.27	0.27	0.54	0	0	0	0	0	0	0				
DRB*27	0	0	0	0	0	0	0.27	0.27	0.80	0	0	0	0	0.27	0	0			
DRB*28	0	0	0	0.54	0	0.27	0	0	0	0	0	0	0	0	0	0	0		
DRB*29	0	0	0	0.54	0	0.27	0	0.27	0	0	0	0	0	0	0	0	0	0	
DRB*30	0.27	0	0	0	0	0	0.27	0.27	0.27	0	0	0	0	0	0	0	0	0	0

Table S5. *MHC-DOB* alleles for both the extended sequence (360 bp) and exon 2 (270 bp) found in our white-tailed deer population and their frequencies. ([#] and ^ indicates that these alleles translated into the same amino acid sequence)

***MHC-DOB* extended sequence**		***MHC-DOB* exon 2**		
Allele	Frequency (%)	Allele	Nucleotide Frequency (%)	Amino Acid Frequency (%)
Odvi-DOB*01	0.53	Odvi-DOB*010211_exon2	14.61	73.42[#]
Odvi-DOB*02	1.32			
Odvi-DOB*11	12.76			
Odvi-DOB*03	10.00	Odvi-DOB*0310_exon2	20.53	73.42[#]
Odvi-DOB*10	10.26			
Odvi-DOB*04	11.84	Odvi-DOB*04_exon2	11.71	20.53^
Odvi-DOB*05	8.82	Odvi-DOB*05_exon2	8.82	20.53^
Odvi-DOB*06	7.50	Odvi-DOB*0607_exon2	10.39	73.42[#]
Odvi-DOB*07	3.03			
Odvi-DOB*08	27.89	Odvi-DOB*08_exon2	27.89	73.42[#]
Odvi-DOB*09	6.05	Odvi-DOB*09_exon2	6.05	6.05

Table S6. Number of nucleotide (below diagonal) and amino acid (above diagonal) differences between the extended *MHC-DOB* sequence (360 bp) alleles for white-tailed deer. The amino acid differences correspond to the amino acid differences seen in *MHC-DOB* exon 2 (Table 7).

	DOB*01	DOB*02	DOB*03	DOB*04	DOB*05	DOB*06	DOB*07	DOB*08	DOB*09	DOB*10	DOB*11
DOB*01	-	0	0	1	1	0	0	0	1	0	0
DOB*02	2	-	0	1	1	0	0	0	1	0	0
DOB*03	2	2	-	1	1	0	0	0	1	0	0
DOB*04	3	3	1	-	0	1	1	1	2	1	1
DOB*05	2	2	2	1	-	1	1	1	2	0	0
DOB*06	3	3	1	2	3	-	0	0	1	0	0
DOB*07	4	4	2	3	4	1	-	0	1	0	0
DOB*08	2	2	2	3	2	3	4	-	1	0	0
DOB*09	3	3	3	4	3	4	5	1	-	1	1
DOB*10	3	3	1	2	3	2	3	3	4	-	0
DOB*11	1	1	1	2	1	2	3	1	2	2	-

Table S7. Number of nucleotide (below diagonal) and amino acid (above diagonal) differences between *MHC-DOB* exon 2 (270 bp) alleles for white-tailed deer.

	DOB*010211_exon2	DOB*0310_exon2	DOB*04_exon2	DOB*05_exon2	DOB*0607_exon2	DOB*08_exon2	DOB*09_exon2
DOB*010211_exon2	-	0	1	1	0	0	1
DOB*0310_exon2	1	-	1	1	0	0	1
DOB*04_exon2	2	1	-	0	1	1	2
DOB*05_exon2	1	2	1	-	1	1	2
DOB*0607_exon2	2	1	2	3	-	0	1
DOB*08_exon2	1	2	3	2	3	-	1
DOB*09_exon2	2	3	4	3	4	1	-

Table S8. Genotype frequencies (%) for the extended *MHC-DOB* sequence (360 bp) alleles in our white-tailed deer population.

	DOB*01	DOB*02	DOB*03	DOB*04	DOB*05	DOB*06	DOB*07	DOB*08	DOB*09	DOB*10	DOB*11
DOB*01	0										
DOB*02	0	0									
DOB*03	0.26	0	0.79								
DOB*04	0.26	0.26	1.58	2.37							
DOB*05	0	0	2.11	1.58	0.79						
DOB*06	0	0	1.84	1.32	3.16	0.53					
DOB*07	0	0.26	0.53	0.53	0.53	0.53	0				
DOB*08	0.26	1.32	5.53	8.68	4.74	2.37	1.05	7.90			
DOB*09	0	0	0.79	0.79	0.26	0.79	1.58	3.16	1.05		
DOB*10	0	0	2.11	0.79	1.84	1.84	0.53	7.11	1.32	2.11	
DOB*11	0.26	0.79	3.68	3.16	1.84	2.11	0.53	5.79	1.32	0.79	2.63

Table S9. Genotype frequencies (%) for *MHC-DOB* exon 2 (270 bp) alleles in our white-tailed deer population.

	DOB*010211_exon2	DOB*0310_exon2	DOB*04_exon2	DOB*05_exon2	DOB*0607_exon2	DOB*08_exon2	DOB*09_exon2
DOB*010211_exon2	3.68						
DOB*0310_exon2	4.74	5.0					
DOB*04_exon2	3.68	2.63	2.11				
DOB*05_exon2	1.84	3.95	1.58	0.79			
DOB*0607_exon2	2.90	5.0	1.84	3.68	0.79		
DOB*08_exon2	7.37	12.63	8.68	4.74	3.42	7.90	
DOB*09_exon2	1.32	2.11	0.79	0.26	2.37	3.16	1.05

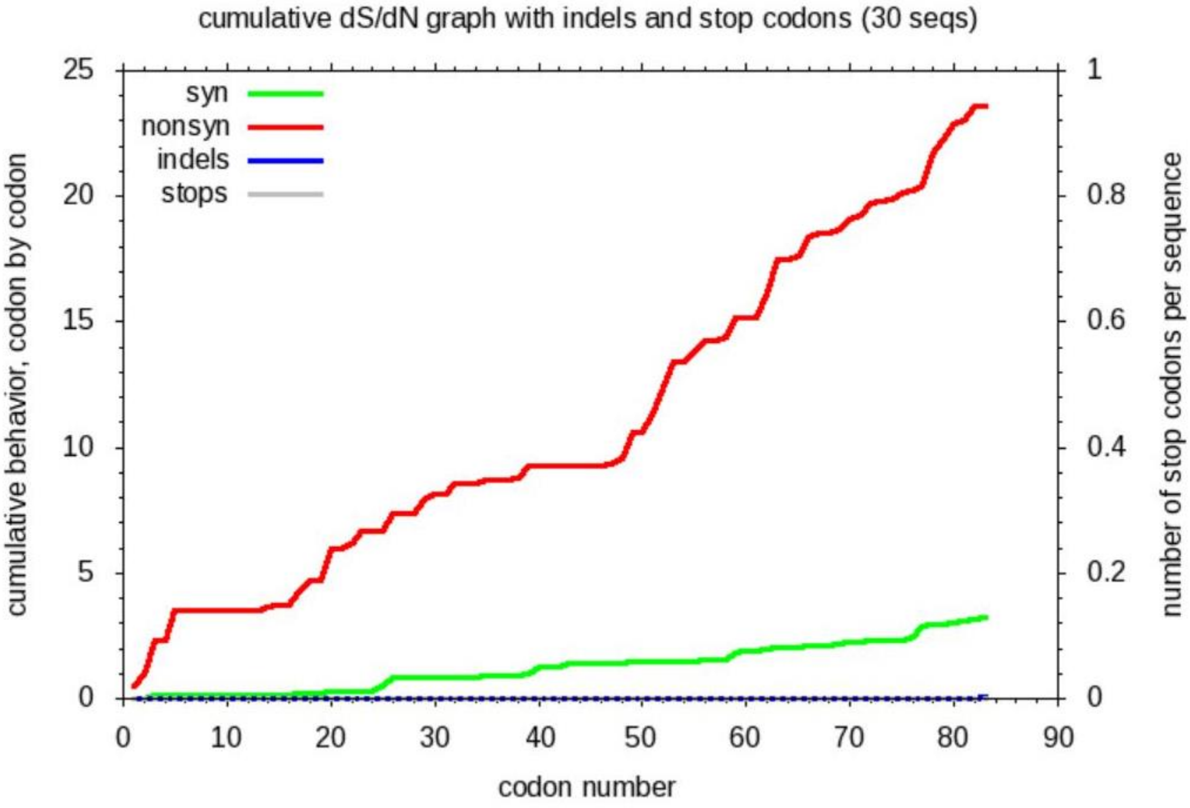

Figure S1. Cumulative mean codon-by-codon ratio of synonymous to nonsynonymous substitutions (dS/dN) for *MHC-DRB* exon 2. Nonsynonymous substitutions are significantly more common than synonymous substitutions for *MHC-DRB* exon 2 in white-tailed deer

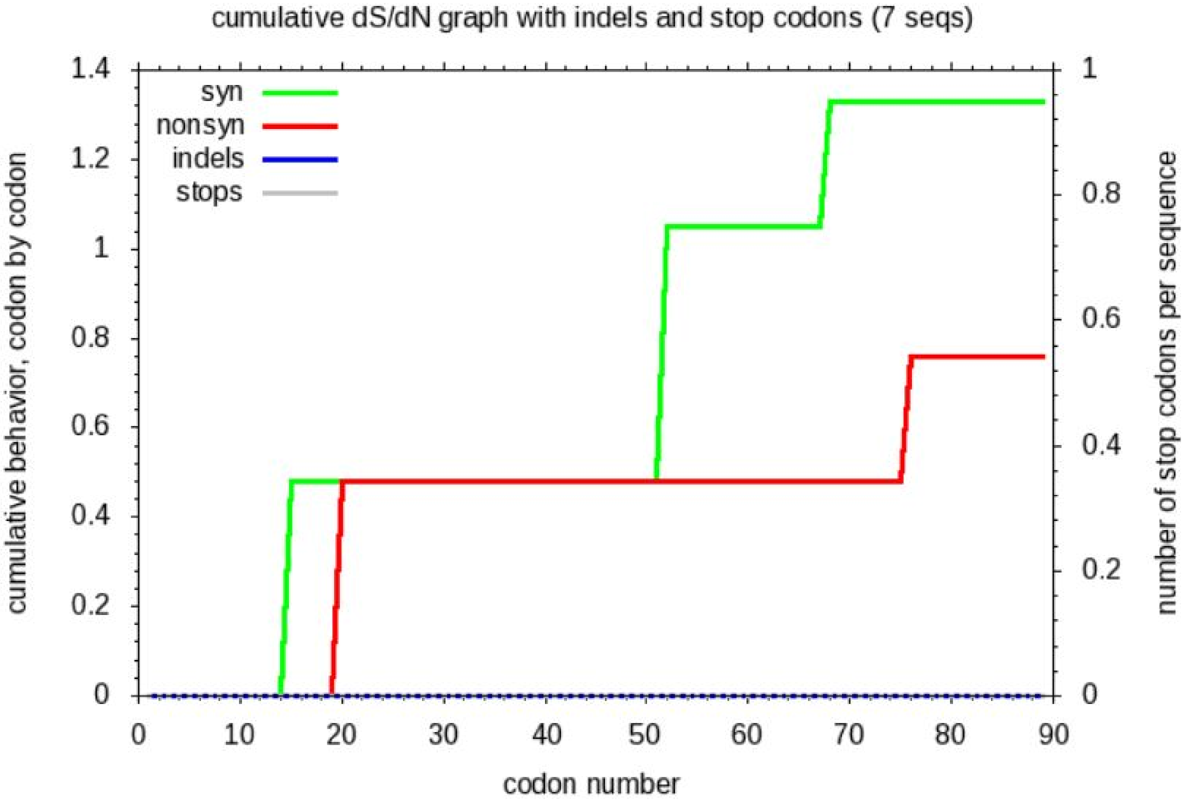

Figure S2. Cumulative mean codon-by-codon ratio of synonymous to non-synonymous substitutions (dS/dN) for *MHC-DOB* exon 2. Synonymous substitutions are overall more common than nonsynonymous substitutions for *MHC-DOB* exon 2 in white-tailed deer, though both are quite rare
